# The consumer footprint: Monitoring sustainable development goal 12 with process-based life cycle assessment

**DOI:** 10.1016/j.jclepro.2019.118050

**Published:** 2019-12-10

**Authors:** Serenella Sala, Valentina Castellani

**Affiliations:** European Commission - Joint Research Centre, Via E. Fermi 2749, 21027, Ispra, Italy

**Keywords:** SDG 12, Sustainable production and consumption, Environmental impact assessment, LCA, Household consumption, Consumer footprint

## Abstract

Sustainable and responsible production and consumption are at the heart of sustainable development, explicitly mentioned as one of the sustainable development goals (SDG12). Life cycle assessment, with its integrated holistic approach, is considered a reference method for the assessment of the environmental impact of production and consumption. This paper presents a study on the environmental impacts of final consumption in Europe in five areas of consumption: food, mobility, housing, household goods, and appliances. Based on the selection of a set of representative products to meet food, mobility, housing, and other consumers’ needs, environmental impacts of products are assessed over their full life cycle: from raw material extraction to production, distribution, use, and end-of-life phase. Life cycle inventories of representative products are multiplied by consumption statistics to assess the impact of an average European citizen in 2010 and 2015. Impacts are assessed considering the sixteen impact categories of the Environmental Footprint method. Results reveal that food is the most relevant area of consumption driving environmental impacts. Use phase is the most important life cycle stage for many impact categories, especially for the areas of consumption housing, mobility, and appliances. For the areas of consumption food and household goods, the most important life cycle phase is related to upstream processes, which corresponds to agricultural activities for food and manufacturing of products components for household goods. Apart from the results, the paper includes a detailed discussion on further methodological improvements and research needs to make use of the Consumer Footprint as an indicator for monitoring SDG 12 and for supporting sustainable production and consumption policies.

## Highlights

• A method for assessing the Consumer Footprint using life cycle assessment and covering 16 environmental impact categories is proposed.
• Impacts of five areas of consumption are assessed: food, mobility, housing, household goods, and appliances.
• The indicator allows for assessing impacts of changes in consumption over time.
• Food is the area of consumption driving the overall impact for several impact categories.
• The indicator may be useful to monitor the Sustainable Development Goal 12 (responsible production and consumption).

## Graphical abstract

**Figure:**
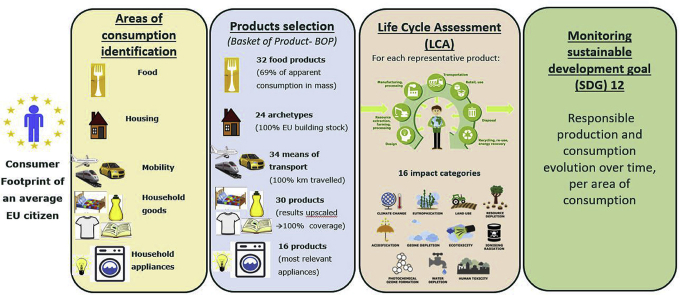

## 1 Introduction

Assessing the environmental impact due to production and consumption of goods and services is a crucial step towards achieving the sustainable development goals (SDGs) (United Nations, 2015) related to responsible production and consumption (SDG 12) and ensuring the achievement of the other SDGs. The production and consumption of goods and services (e.g., final household consumption) are essential contributors to environmental impacts. With the “One Planet” network (UN, 2019), the United Nations (UN), is bringing together actors from all regions and all sectors to ensure a shift to more sustainable modes of production and consumption.

In order to move towards more sustainable consumption patterns, the current consumption trends and their global environmental impact have to be analysed and monitored. This study is focusing on the European context, as European Union has committed to play a pivotal role in SDGs achievements (e.g., in EC, 2016a). The study is serving not only the need for monitoring the SDG 12 but as well respond to the request of the 7th European Environmental Action Program (EAP) (EU, 2013) which required to develop methods for measuring and benchmarking resource efficiency and related impacts, including considering greenhouse gases emissions, land use, water and material use and for assessing the appropriateness of the inclusion of a lead indicator and associated targets.

Final consumption in the European Union constituted at least half of the Gross Domestic Product (GDP) in nearly three quarters (20) of the European Union (EU) Member States in 2016 (ranging from 28% in Luxembourg to 69% in Cyprus). More than half of the EU-28 household expenditure was spent on housing (24%), transport (13%), and food (12%). These consumption categories were identified earlier as those that contribute the most to the environmental impact of the consumption (EC - JRC, 2012a; EEA, 2012; EEA, 2013). However, other areas of consumption of goods, such as household goods and appliances (such as fridges, televisions, cooling devices etc), are in the rapid expansion (representing respectively 11% and 6% of household expenditure) and deserve to be considered and assess in terms of their environmental implications.

Over the years, various methods for estimating global environmental pressures and impacts due to consumption patterns of households and governments have been developed. Those methods addressed both direct and indirect impacts. Based on the literature review conducted in the EIPRO (Environmental Impact of Products) study (Tukker et al., 2006) and in EC-JRC (2010), three main typologies of studies assessing environmental pressures and impacts associated with consumption have been identified by the authors: top-down, bottom-up, and hybrid approaches. All these approaches may be applied either accounting for environmental pressures (e.g., emissions or resource uses) or for assessing environmental impacts, as in life cycle assessment (LCA). For example, Lutter and Giljum (2014), discriminate i) bottom-up approaches into methods based on process-based LCA and those which are not, (such as economy-wide material flow analysis (MFA)) (Eurostat, 2012a, b; Eurostat, 2013) and ii) top-down, national footprint accounts, which usually focus on one area of concern at the time, e.g. land footprint (Bruckner et al., 2015), water footprint (Hoekstra and Mekonnen, 2012), that may be ultimately considered together in a footprint family (Galli et al., 2012). However, moving from pressure-based indicators such as those of MFA and the so-called “footprint family”, towards impact-based indicators as in traditional process-based LCA is of utmost importance (Ridoutt et al., 2016).

The literature on the application of top-down approaches based on input-output is rapidly expanding (see, e.g. the special volume on input-output assessment based on Exiobase 3 (Tukker et al., 2018)). Many studies are addressing the impacts at European scale. However, despite their capability of assessing impacts at macro scale encompassing all economy-wide flows, the top-down methods lack details and realism in representing specific economic behaviour as well as physical mass balances at the product level. In fact, physical exchanges occurring between economic sectors and and final consumers are approximated through average products, characterized by average price, average mass and average environmental profile, although such average product is usually a virtual one. Generally this approximation might hold for relatively homogeneous sectors. That is not the case for largely heterogeneous sectors in which the diversity of commodities produced is usually very high. Moreover, the analysis of main contributors of impacts, in terms of production and consumption phases and specific processes (e.g., associated with different life cycle stages), is not possible.

Hence, bottom-up approaches may be considered a key to assess sustainable production and consumption increasing the level of detail and discriminating better between the contributions to impacts due to different products in the same areas of consumption. However, despite bottom-up studies at the level of a single product are numerous, hybrid and bottom-up approaches addressing the consumption comprehensively and modelling multi-products at the macro-scale (e.g.at a continental scale) are still relatively limited.

In the literature, bottom-up studies addressing the impact of consumption at macro scale are based on life cycle assessment (LCA) conducted for specific representative products, which are then up-scaled to overall consumption figures through several up-scaling techniques (e.g., EC-JRC, 2012a; EC-JRC, 2012b; Notter et al., 2013). There are as well studies focusing on citizen lifestyles (Kalbar et al., 2016; Froemelt et al., 2018), which nonetheless focus on one nation, and lack an European perspective.

The study presented here builds on the basket of product indicators project conducted by the European Commission - Joint Research Centre (EC- JRC) since 2010 (EC-JRC, 2010b, EC-JRC, 2010a; EC-JRC, 2012a, EC-JRC, 2012b). The study aims at comprehensively assessing different areas of consumption with a life cycle-based bottom-up approach at the scale of the entire Europe. The rationale is to build a baseline set of indicators to monitor the impacts of consumption patterns over time and to run scenarios against the baseline to test the extent to which different innovations or intervention are helping in reducing the impacts.

The novelty of the present study stems from four different aspects: (i) the use of bottom-up approach to assess impacts of consumption at macro scale by means of representative products modelled to reflect average consumption patterns in EU; (ii) the assessment of the evolution of the impacts over time, matching consumption statistics; (iii) the development of a baseline scenario useful to test the potential effect of eco-innovation in different areas of consumption; (iv) an outlook on the use of these indicators for monitoring the SDG 12, adopting process-based LCA.

## 2 Method

The Consumer Footprint assesses the potential environmental impacts coming from household consumption, by means of process-based LCA of goods and services purchased and used by a certain entity, e.g., the entire European population.

The analysis of the environmental impacts has been performed separately for five consumption areas, using the approach of the basket of products (BoP), as well as the sum of the areas of consumption. The basket of product approach combines the information about the structure and intensity of the final consumption with the life cycle inventories of products consumed in order to calculate the environmental impact profile of the final consumption of an average European Union citizen (EC-JRC, 2012a; Sala et al., 2019). These environmental impacts refer to the entire life cycle of the chosen basket of goods and services per each area of consumption.

For the calculation of the Consumer Footprint, five critical areas of household consumption were considered: housing, mobility, food, household goods, and appliances. These areas were selected based on relevance in terms of mass and value consumed in Europe and due to the potential impact associated with their supply chains. For each area of consumption, a process-based life cycle inventory (LCI) model for a basket of products was built.

It is important to point out that the products included in the baskets are only a subset of total consumption. Therefore, the Consumer Footprint provides an index for monitoring and analysis, and not an absolute measure of environmental impact per person.

In order to develop each BoP, the following steps were performed:

• Quantitative and qualitative analysis of the structure of the European Union consumption in the selected areas of consumption – for the years 2010 and 2015 – including analysis of international trade to model the phases of the supply chains associated with imported goods.
• Selection of a BoPs representative for the structure of consumption, and development of a detailed list of the available datasets for the identified products with the feasibility assessment of their application for the purpose of the average EU consumption evaluation.
• Modelling of process-based LCI models for the selected representative products, which included:
  o Modelling of the supply chain of the representative products based on analysis of statistical data related to the specific product category.
  o Modelling of the use phase to represent average usage conditions in the European Union
  o Modelling of the End of Life (EoL) stage including benefits and burdens of recycling and waste treatments.
• Calculation of the environmental impacts for each of the basket as well as per citizen.

To assess the environmental impacts, models and factors recommended for Environmental Footprint as in the EF package 2.0 (EC, 2018) were used as a life cycle impact assessment (LCIA) method. This includes:

• the characterization of impact in 16 impact categories (climate change, acidification, ozone depletion, eutrophication -terrestrial, marine, and freshwater, photochemical ozone formation, particulate matter, ionizing radiation, ecotoxicity, human toxicity cancer and – non cancer, land use, water use, resource use -metal and minerals, and fossil)
• the normalization using global references (Sala et al., 2017; Crenna et al., 2019a)
• the weighting (Sala et al., 2018) used for calculating a single score Consumer Footprint (see S1 for details).

Quantitative and qualitative analysis of the environmental impacts of each basket, including contribution analysis in terms of most relevant products, most relevant processes, most relevant life cycle stages, and most important emission/resource use driving the overall impact per impact category.

Finally, the inventories of the five BoPs were added up to calculate the Consumer Footprint in EU, namely the environmental profile of household consumption in Europe.

To ensure consistency among the five BoPs - food, mobility, housing, household goods, and appliances - and to allow summing the impacts to calculate the Consumer Footprint in EU, some general principles have been applied, as follows:

• The reference year was 2010. Additional analysis for the year 2015 was performed, to assess evolution of impacts due to changes in household consumption.
• Scope of the analysis was the final household consumption in the EU-27 for one year.
• Results are presented for the total EU-27 and for an average European citizen.
• Eurostat was the primary source for data about consumption and trade of the products.
• The definition of system boundaries was aimed at including all the relevant activities for each area of consumption. When summing the BoPs, overlaps among areas of consumption were removed, to avoid double counting.
• The cradle-to-grave approach was applied. The life cycle stages considered for each basket were upstream activities (e.g., agricultural phase for the BoP food and manufacture of product components for BoP household goods), production, packaging, distribution and logistics, use phase, maintenance, and end of life (EoL). Those phases were adjusted to the specific features of each basket when needed (as illustrated in Table 1). The EoL was modelled in a modular way, which allows accounting for burdens and benefits of recycling detached from the rest of EoL, if needed. Where relevant, burdens were allocated over the lifespan of the product.

**Table 1:** Life Cycle phases and activities included in the five BoPs composing the Consumer Footprint indicator.

Life Cycle phase	BoP Housing	BoP Mobility	BoP Food	BoP Household goods	BoP Appliances
Upstream	Production of construction materials	Construction of mobility infrastructure (roads, railway, airports)	Agricultural activities: cultivation of crops and animal rearing	Manufacture of product components	Manufacture of product components
Production	Construction of the building	Vehicle production	Processing of ingredients; slaughtering and processing of meat; chilled or frozen storage of products before distribution	Manufacture of final product	Manufacture of final product
Packaging	*(not applicable)*	*(not applicable)*	Production of packaging materials; EoL of packaging materials	Production of packaging materials; EoL of packaging materials	Production of packaging materials; EoL of packaging materials
Logistics	*(not applicable)*	*(not applicable)*	Storage at distribution centre (whenever relevant); transport from production site to retail; storage at retail (for refrigerated products)	Transport to retail	Transport to retail
Use	Energy and water use by dwelling's users; wastewater treatment	Production of fuel; emissions related to vehicle use (e.g. from combustion of fuel in internal combustion engines); tire wear emissions, brake wear emissions; road wear emissions.	Transport of product from retail to user; cooking (whenever relevant), refrigerated storage of products at home	Transport of product from retail to user; electricity use (whenever relevant); water use (e.g. for detergents);	Transport of product from retail to user; electricity use; water use (e.g. for dishwashers and washing machines), wastewater treatment
Maintenance	Production of components substituted during the building lifetime (e.g. windows); EoL of substituted components	Production of components substituted during the vehicle lifetime (e.g. tires); EoL of substituted components	*(not applicable)*	*(not applicable)*	Production of components substituted during the appliance lifetime; EoL of substituted components
EoL	Building demolition; sorting of materials; treatment of sorted materials (including recycling)	Vehicle demolition; sorting of materials; treatment of sorted materials (including recycling)	Final disposal of food waste; wastewater treatment and auxiliary processes due to toilet use	Wastewater treatment (for detergents, personal care products, and toilet paper)	Wastewater treatment (for washing machines and dishwashers)

Notwithstanding a common assessment framework, the inherent differences in the five product categories (e.g., the complexity, the lifetime, and the multiple functions of some products such as dwellings) imply necessarily to differentiate – within the general framework – the assumptions and the methodological choices made for each specific BoP.

The most relevant differences occur in the selection of the representative products for each basket, due to the need of considering the specific characteristics of each product category, such as the site-dependent features (e.g. energy use due to different climate zone for housing and appliances) or the usage-dependent ones (e.g. highway, urban or rural roads for road transport). Table 2 illustrates the main differences in the calculation steps of the five BoPs.

**Table 2:** Key characteristics of the five BoPs and assumptions adopted for their calculation.

	Food	Mobility	Housing	Household goods	Appliances
Functional Unit (F.U.)	Average food consumption by an EU citizen in one year (including food losses at each stage)	Average passenger-kilometers per EU citizen for the means of transport considered	Average consumption related to housing (only for permanent occupied dwellings) for an average EU citizen during one year	Average purchase and use of household goods by an EU citizen in one year	Average purchase and use of household appliances by an EU citizen in one year
Lifetime of products	1 year	Different for each vehicle type. 150 000 km for cars, 40 years for trains, ad according to ecoinvent assumptions for buses and airplanes	100 years	1 year for all products except furniture (10 years) and mattresses (10 years)	Different for each product, spanning from 2 years (incandescent lamp) to 19 years (electric oven)
Foreground data sources	Literature	Eurostat	Eurostat, national statistical data and literature	Ecolabel background studies (EC-JRC, 2017) and literature	Ecodesign preparatory studies (EC-JRC, 2017) and literature
Background databases	Agrifootprint, ecoinvent 3	ecoinvent 3	ecoinvent 3	ecoinvent 3	ecoinvent 3
Criteria for the selection of representative products	- Mass quantity and economic value of food consumed - Inclusion of food products that are known to have significant environmental impact (e.g. chocolate) - Emerging consumption trends (e.g. bottled water and preprepared meals)	Contribution to final energy consumption due to mobility in the EU27.	Definition of archetypes of dwellings (Lavagna et al., 2018) based on: - Dwelling types and building typologies - Climatic zones in the EU - Year of building construction	- Products covered by Ecolabel and Green Public Procurement (GPP) criteria - product groups for which a Product Environmental Footprint (PEF) pilot was ongoing	Products representing 3 main types of appliances: - white goods (e.g. fridge); - appliances for basic functions related to the housing (e.g. space cooling); - appliances for entertainment and leisure. Priority is given to energy-related products covered by the Ecodesign directive (EC, 2009)
Number of representative products selected	32 food products representing 15 food categories	27 means of transport (12 types of Gasoline passenger cars, 8 types of Diesel passenger cars, LPG passenger car, 3 types of 2wheelers, 3 types of buses, 2 types of trains, 3 types of flights)	24 representative dwellings in three climatic zones (12 for single family houses and 12 for multi-family houses)	30 products representing 8 product groups, namely : household detergents, hygiene products, soaps and shampoos, furniture, bed mattresses, footwear, textile, paper based products	16 products representing 9 types of appliances, pertaining to the following product groups: dishwashing, washing and drying machine, refrigerators, air conditioning, cooking appliances, lighting, computer, and television.

A brief description of the modelling approach undertaken for each BoP is provided in the following and in supplementary information (section S2), while extensive details on data sources and modelling assumptions are illustrated in specific publications on: BoP Housing (Lavagna et al., 2018; Baldassarri et al., 2017), BoP Mobility (Castellani et al., 2017a), BoP Food (Notarnicola et al., 2017; Castellani et al., 2017b and the extension provided by Crenna et al., 2019b), BoP Appliances (Reale et al., 2019; Hischer et al., 2019), and BoP Household goods (Castellani et al., 2019a).

In the case of the BoP Food and the BoP Household goods, household consumption in EU-27 was represented by apparent consumption, calculated as the number of goods produced in Europe, plus the number of goods imported, minus the number of goods exported (Eurostat, 2017a).

In the case of BoP Appliances, the number of representative products included in the functional unit (in pieces) was calculated starting from the analyses of the existing stock, performed in the context of the Ecodesign preparatory studies (EC-JRC, 2017). The reason of this choice was twofold. Firstly, all the selected appliances have a service life longer than one year and this affects the annual apparent consumption. Secondly, all appliances consume energy during their service life. Consequently, including in the BoP the apparent consumption would not capture the realistic environmental impacts due to the annual purchase and use of appliances. Hence, a different approach was followed. For each product, the whole stock present in European households was allocated to the reference year (dividing it by the number of service life years of the representative product chosen, as defined in the Ecodesign preparatory studies, EC-JRC, 2017). Similarly, the BoP Housing included the entire European building stock in 2010, represented by 24 building archetypes, and allocated to one year by dividing the inventory by the lifetime of buildings (assumed to be 100 years, according to Lavagna et al., 2018). Data about the characteristics of the building stock were retrieved from the results of several Intelligent Energy Europe (IEE) Projects (ENTRANZE, 2014; ODYSSEE, 2014; TABULA, 2012; and EPISCOPE, 2012). Finally, the functional unit of the BoP Mobility was the number of kilometers travelled by EU-27 citizens in one year, with several means of transport (cars, 2-wheelers, buses, trains or airplanes). Data about the intensity of use were taken from the Eurostat database (Eurostat, 2017b).

The Life Cycle Inventories (LCIs) of the representative products were in line with the International Life Cycle Data system (ILCD) guidelines (EC-JRC, 2010b). The LCI followed, to the extent it was possible and relevant, the Environmental Footprint methods and guidelines as published in the European Commission (EC) Communication “Building the Single Market for Green Products” (EC, 2013a) and related technical documents (e.g. EC, 2013b; EC, 2018). The primary data sources used for building the inventories were ecoinvent v.3.2 (BoPs housing, mobility, food, household goods, and appliances) and Agri-footprint v.2 (BoP food) databases, complemented with data from the scientific literature.

In order to build the inventory of each BoP regarding the functional unit considered (i.e., consumption in EU-27 in 2010), the LCIs obtained for each representative products were then multiplied by the amount of that product that was consumed in one year by EU-27 citizens.

Regarding BoPs Food and Household Goods, the calculation approach is as follows:

$$BoP\;Food\;\left(or\;Household\;goods\right)=\overset{n}{\underset{RPs}{\sum }}\text{Mass of apparent consumption*LCI of RPs}$$

where RPs are the representative products.

Regarding BoP Appliances, the calculation approach is as follows:

$$BoP\;Appliances=\overset{n}{\underset{RPs}{\sum }}\left(\frac{N{}^{\circ}\;of\;units\;of\;RPs\;in\;the\;EU\;stock}{N{}^{\circ}\;of\;service\;life\;years\;per\;product}\right)*LCI\;of\;RPs$$

Regarding BoP Mobility, the calculation is based on the amount of km travelled by European citizens by each mean of transport (namely car, two-wheelers, buses, train, airplane), as follows:

$$BoP\;Mobility=\overset{n}{\underset{RMTs}{\sum }}\text{Km travelled per year per means of transport*LCI of RMTs}$$

where RMTs are the representative means of transport.

Finally, regarding BoP Housing, the calculation is based on the allocation of the total amount of existing buildings in Europe to the 24 dwelling archetypes, as follows:

$$BoP\;Housing=\overset{n}{\underset{RDAs}{\sum }}\text{N}{}^{\circ}\text{ of dwellings in each archetype*LCI of RDAs}$$

where RDAs are the representative dwelling archetypes.

Details on the representative products or archetypes selected for each basket, the respective quantities considered to represent household consumption in Europe in the reference year 2010 and the updated quantities for 2015 are reported in the supplementary material (section S2).

### 2.1 Summing up the five BoPs

When summing up the five BoPs to calculate the Consumer Footprint in the EU, two issues were considered. Firstly, there were activities potentially overlapping among BoPs (especially regarding the use phase). The overlapping is not a problem when considering each BoP separately, but can lead to double counting when summing all BoPs. Therefore, for the calculation of the Consumer Footprint in Europe, overlapping activities were identified in all BoPs and then they were consistently included only in the BoP with the broader scope and excluded from the others, to avoid double counting. Table S3.1 reports details of the overlaps identified and of the decision taken about their inclusion in one of the BoPs for the sum.

Secondly, to assess the impact of household consumption in EU, each BoP should take into account, to the maximum extent possible, the total consumption in the area under investigation. In fact, the representativeness of the products selected differed for the five BoPs.

For BoP Housing, the 24 archetypes (representative dwellings) selected represented the entire European building stock in the reference year 2010 (i.e., all the existing buildings were allocated to one specific archetype). The resource and global impacts of buildings are distributed over the service life, allocating a share for each year. Details on this aspect are provided in Lavagna et al., (2018).

The BoP Mobility considered 100% of the kilometers travelled by European citizens with private means of transport (private cars and 2-wheelers), 100% of the air transport, and 100% of rail transport. Tram and metro, as part of urban public transport, were not modelled in the BoP Mobility. Also, marine passenger transport was excluded because Eurostat database did not provide statistics on the share of those means of transport over the total km travelled by European citizens. However, according to the Statistical Pocketbook on transport (EC, 2016b), tram and metro contributed to 1.5% of all km travelled by European citizens in 2010. Similarly, marine passenger transport accounted for only 0.4% of all passenger transport in Europe in 2010. Therefore, the means of transport included in the BoP Mobility allow assessing the impact of about 98% of the km travelled in Europe with private and public means of transport.

The other BoPs were built using representative products, so they did not include 100% of the products consumed in the three areas of consumption described (food, household goods, household appliances). Therefore, quantities of representative products in BoP Food, BoP Household goods and BoP Appliances have been upscaled to enlarge the representativeness of these two baskets in the assessment of the Consumer Footprint in EU, as in the following equation:

Consumer Footprint=BoP Housing+BoP Mobility+BoP Food∗UF+BoP Household goods∗UF+BoP Appliances∗UF

where UF is the upscaling factor.

For example, the three representative products under the product group “Meat” in the BoP Food (76.7 kg/person*yr^−1^ in total) represents 89% of the quantity of meat purchased by European citizens in one year (86.4 kg/person*yr^−1^). Therefore, the quantity of the three meat products included in the basket is up-scaled to 100% (i.e., to 86.4 kg/person*yr^−1^) to represent the total amount of meat consumed (i.e., including also other types of meat, not modelled in the BoP). It is important to remark that because of the upscale, the representativeness of BoP Food, BoP Appliances and BoP Household goods is 100% for the product groups considered, but still, they do not cover 100% of the overall food, appliances, and household goods consumption. In fact, there may be other product groups that are part of those areas and that are not included in the BoP.

### 2.2 Assessing changes in the impacts of consumption between 2010 and 2015

The BoPs are meant to represent household consumption in Europe in a reference year in five main areas of consumption (housing, mobility, food, appliances and household goods). The baseline scenarios calculated for the five BoPs refer to the baseline year 2010, and, consequently, to EU-27 (Croatia entered in 2013).

To monitor the evolution of the impact over time, the consumer footprint has been calculated as well for the year 2015, taking into account that EU moved from EU-27 to EU -28 (with the inclusion of Croatia), the overall increase of EU population size (+1.19%), and the evolution of the consumption patterns. This analysis is conducted for each of the five BoPs considered.

Consequently, the reference population is updated (from 502 million citizens in 2010 in EU-27 to 508 million citizens in 2015 in EU-28) and the apparent consumption of representative products, is calculated for 2015 as it was done for the reference year 2010. As a general assumption, the technical features and the performance of appliances and household goods and the characteristics of the food production chain for food products are kept constant from 2010 to 2015. On the contrary, in the case of BoP Mobility and BoP Housing, the update also includes some modifications in the structure of the baseline model, to account for changes in the composition of the building stock and the mobility fleet. More in details, dwellings built between 2010 and 2015 are added in the BoP Housing, whereas electric and hybrid cars (which were not significantly used in 2010 but became relevant in 2015) are added to the EU mobility fleet, as well as Euro 6 cars.

## 3 Results and discussion

Building on the method described above, the results of the Consumer Footprint are presented:

(i) separately for the different baskets, as total values and per person,
(ii) summed up, removing possible double counting,
(iii) as for changes over the period 2010–2015.

The environmental impacts are reported for 16 environmental impact categories and a contribution analysis is performed at three levels: contribution of products to the overall impact, contribution of processes, and contribution by the elementary flow.

The discussion on the implications of methodological aspects on the interpretation of the results is reported in section 3.3.

### 3.1 Environmental impact of consumption in the five baskets of products

**Fig. 1:**
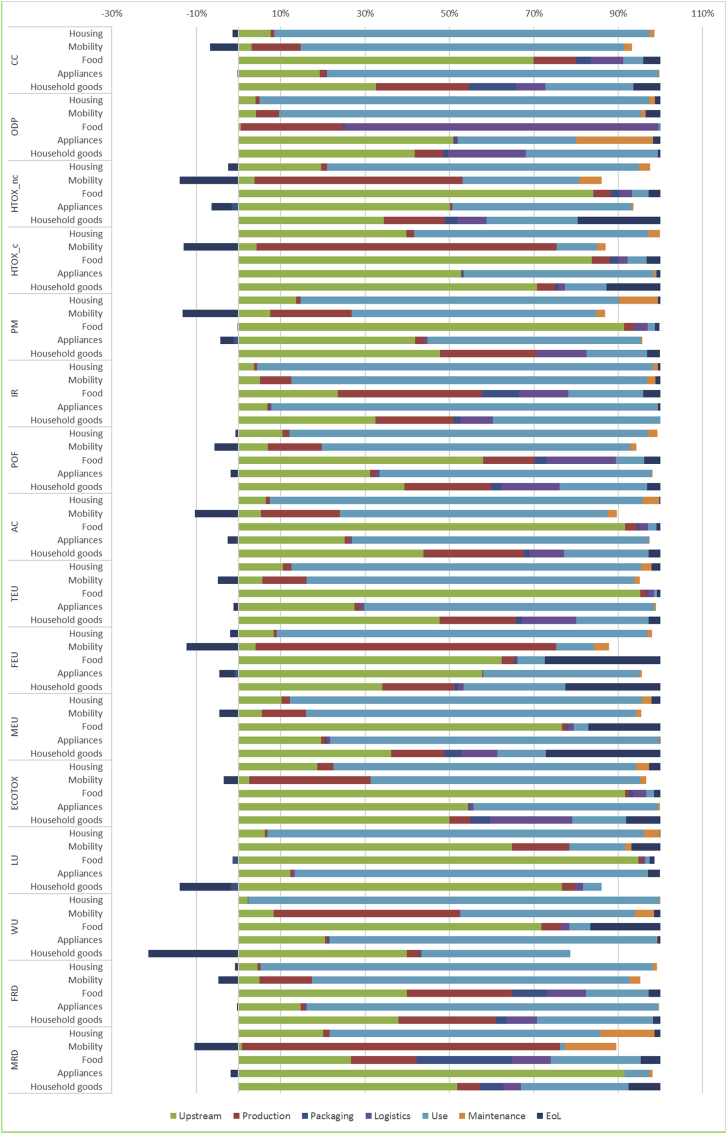
Overview of the impacts of the different life cycle stages of the five considered consumption categories with their relative contribution (%) to the overall impact.

The overall results of the Consumer Footprint in EU split into the five specific BoPs (food, housing, mobility, household goods and appliances) are reported in Table 3. Results per person are reported in S4. Details of the calculations of each BoP are available in the related reports (Baldassarri et al., 2017; Castellani et al., 2017a, 2017b, 2019a; Reale et al., 2019). In Fig. 1, a comprehensive overview of the results illustrating the relevance of the life cycle stages of the considered consumption categories is provided.

**Table 3:**
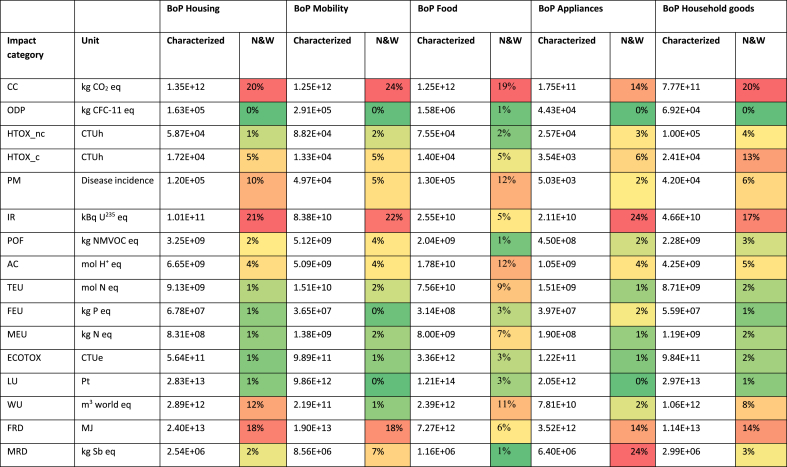
Summary of the Consumer Footprint results of the baseline scenarios (2010) for the five BoPs. A colour code is used to show weighted results, from red (highest value) to green (lowest value).

N&W: Normalized and weighted results, % of relevance of the impact categories. Acronyms for impact categories: CC: climate change; ODP: ozone depletion; HTOX_c: human toxicity, cancer; HTOX_nc: human toxicity, non cancer; PM: particulate matter; POF: photochemical ozone formation, human health; IR: ionising radiation; WU: water use; ECOTOX; freshwater ecotoxicity,; CC: climate change; FRD: resource use, fossil; ODP: ozone depletion; MEU: eutrophication, marine; FEU: eutrophication, freshwater; LU: land use; TEU: eutrophication, terrestrial; AC: acidification terrestrial; MRD: resource use, minerals and metals. For the indicators units: CTUh: comparative toxic units for human health; CTUe: comparative toxic units for freshwater ecotoxicity; NMVOC: non methane volatile organic compounds; Pt: point (dimensionless).

The use phase is the most relevant life cycle stage for many impact categories, especially for the areas of consumption housing, mobility, and appliances. In the case of housing, the contribution of the use phase is more than 90% for the following impact categories: climate change (91% of the impact of housing), ozone depletion (92%), ionising radiation (94%), water use (97%), and fossil resource depletion (95%). The use phase of mobility (i.e., the operating phase of the means of transport considered) contributes largely to climate change (89%), fossil resource depletion (83%), and particulate matter (79%). The use phase of appliances (which corresponds mainly to energy use) generates 79% of the climate change impacts, 84% of fossil resource depletion and 92% of the impact of ionizing radiation. Of course, the importance of the use phase in determining these impacts may change over time. For example, for the housing, the 91% of contribution to the use phase is related to the modelled building stock (for the year 2010) in which many there is still a predominance of building with very low energy performance. Over time, the evolution of the building stock towards a higher share of buildings with reduced energy consumption may imply that other life cycle stages of the housing become relevant (e.g. the construction).

The impact of food consumption is dominated by the upstream phase, which corresponds to agricultural activities. Its contribution is more than 90% for land use (97%), terrestrial eutrophication (95%), acidification (92%), particulate matter (92%), and freshwater ecotoxicity (92%), and more than 80% for human toxicity (both cancer and non-cancer effects). Upstream activities are relevant also for appliances and household goods. In these cases, they correspond to the extraction of raw materials and the production of components used to manufacture the final products. Upstream activities contribute to 95% of mineral resource depletion, and 63% of freshwater eutrophication for the impact of appliances, whereas they contribute to 80% of land use, 71% of human toxicity, and 70% of water use for the impacts of household goods.

The production phase appears to be particularly relevant for mobility, with contributions higher than 90% to human toxicity (96%), mineral resource depletion (95%). The production phase of mobility includes both the assembly of the means of transport and the production of their components. The production phase has a more limited contribution to the impact of other areas of consumption considered. The only exceptions are for food (34% contribution to ionizing radiation, 25% to fossil resource depletion, and 24% to ozone depletion) and household goods (24% contribution to acidification and 23% to fossil resource depletion).

Packaging has a limited contribution as well. Again, the only exceptions are for food and household goods. Packaging production and packaging EoL contribute to 23% of mineral resource depletion and 9% of ionizing radiation generated by food consumption, and to 11% of climate change generated by the life cycle of household goods.

Logistics is the most critical contributor to ozone depletion for the basket food, due to the emissions of refrigerants used in the cold chains (transport and storage of some food products, such as e.g. meat and dairy products). The transport of household goods contributes to 19% of freshwater ecotoxicity and 19% of ozone depletion.

Maintenance activities, included in the life cycle of housing, mobility, and appliances, generally have a limited contribution (ranging from 1% to 18%) to all the impact categories considered. The highest contribution is related to the maintenance of appliances, where it generates 18% of the impacts of ozone depletion (18%) due to the substitution of refrigerants in room air conditioners. In the case of housing and mobility, maintenance activities contribute to mineral resource depletion (13% for housing and 15% for mobility), because of the substitution of components in the building (e.g., windows) or in the car (e.g., tires).

Finally, the EoL contributes to some extent in the case of household goods (27% to marine eutrophication, 23% to freshwater eutrophication, and 20% to human toxicity) and food (27% to freshwater eutrophication and 17% to marine eutrophication). The EoL also generates some benefits (up to 20% for water use in the case of household goods), due to the recycling of materials and the avoided production of virgin ones.

### 3.2 Overall environmental impacts resulting from the sum of the different baskets

**Fig. 2:**
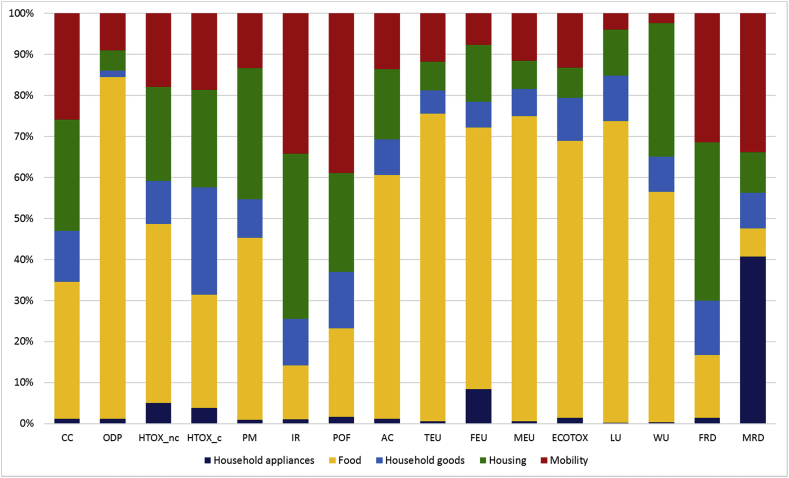
Consumer Footprint in the EU: relative contribution of the five areas of consumption. BoPs results are shown after removing of overlaps. Extended names of impact categories is reported at the bottom of Table 3.

The overall environmental impact resulting from the sum of the five baskets is reported in Table 4. The contribution of each of the five areas of consumption (BoPs) considered is illustrated in Fig. 2. When interpreting these results, it has to be considered that the contribution of each BoP is calculated after removing the overlapping activities. This is particularly relevant for energy use in the use phase, which has been entirely allocated to housing and removed, for instance, from the use phase of appliances. Results of the Consumer Footprint in the EU highlight that food is the area of consumption that contributes the most to 12 impact categories out of the 16 considered. Housing is the second most important contributor in general and the most relevant one for fossil resource depletion and ionizing radiation, whereas mobility is the most relevant one for photochemical ozone formation and the second most important for ionizing radiation, fossil resource depletion, and mineral resource depletion. Household goods and appliances have generally a limited contribution to all the impact categories, except mineral resource depletion, for which appliances are the main contributor (mainly because of the extraction of metals – including precious metals – used in their manufacture). As mentioned before, the relative limited importance of appliances for the other impact categories is also an effect of the removing of energy use in the use phase, to avoid overlaps with housing. However, the identification of food, housing, and mobility as the three most impacting areas of consumption confirms results obtained in previous studies done with an input-output approach (e.g., Tukker et al., 2016).

**Table 4:**
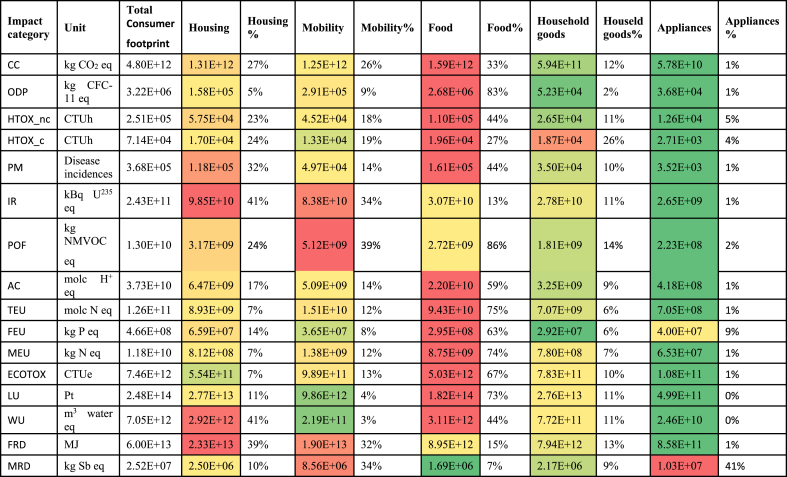
Consumer Footprint in EU: total characterized results and breakdown in the 5 areas of consumption (year 2010). Extended names of impact categories is reported at the bottom of Table 3.

However, differently from previous studies, the calculation of the Consumer Footprint in the EU, allow the identification of the main products responsible of the impacts as well as the life cycle stages, which contribute the most to the impacts.

For Food, meat (especially beef and pork) and dairy products are the most impacting product groups. The main impact for the life cycle of pork and beef meat products comes from the emissions due to production of feed (mainly compound feed, but also grass silage and grass in pasture). Direct emissions from animal husbandry (methane, dinitrogen oxide, ammonia, etc.) contribute as well.

For housing, the higher contribution comes from the building in moderate climates, both single-family houses (SFHs) and multi-family houses (MFHs). These two types of dwelling together represent about 70% of the European building stock and contribute to about 70–80% of the overall impact (30% MFHs and 40% SFHs), depending on the impact category considered. Regarding the mobility, i.e., the modes of transport used by the European citizens, passenger cars are by far the most important ones, in terms of impact generated, compared to the other product groups. Within this group, cars in the engine capacity range 1.4–2.0L, both diesel and gasoline-fuelled, are the most important ones. This is due to a combination of the impact of this type of cars (and especially the fuel consumption in the use phase) and the number of cars in the European fleet belonging to these categories (36% diesel and 23% gasoline).

For household goods, the higher contribution to the overall impacts comes from the following product groups: paper products, detergents, furniture, and clothes, with different shares depending on the impact category considered. This contribution is partly due to the impact of one unit of the products considered and partly to the intensity of the consumption of each product in the BoP.

The greatest contribution to the overall impacts generated by the purchase and use of appliances in EU comes from dishwasher, washing machine, refrigerator, lighting, and TV screen. Again, this contribution is partly due to the inherent properties of the life cycle of the products considered and partly to the amount consumed in the BoP.

As described in section 3.1, some activities are driving the impact of a specific BoP across all or most of the impact categories considered. That holds true for agricultural activities in the case of food, with a contribution higher than 80% across all impact categories. For the housing, heating (either from coal or from wood) is contributing to climate change, human toxicity non-cancer effects, particulate matter, photochemical ozone formation, acidification, terrestrial eutrophication and marine eutrophication. For mobility, emissions from air transport and passenger cars during the use phase (i.e., emissions from fuel burning) contribute to climate change, photochemical ozone formation, acidification, terrestrial eutrophication and marine eutrophication. For household goods, the electricity generation outside Europe (i.e., electricity used in the production of goods, when this phase happens outside Europe) contributes to several impact categories, namely climate change, particulate matter, photochemical ozone formation, acidification, and terrestrial eutrophication.

The contribution of the European electricity mix, i.e., of the electricity used mainly in the use phase of products and services represented in the baskets (and included here mainly in the BoP housing) is contributing to more than 50% of impacts generated on climate change, acidification, terrestrial eutrophication and marine eutrophication.

Other activities appear to be particularly relevant for some impact categories because their contribution is recurrent across the different areas of consumption. This consideration is valid for:

— the contribution of onshore oil and gas production to land use impacts (for housing, mobility, household goods, and appliances)
— the contribution of the treatment of spent nuclear fuel to ionizing radiation (for housing, household goods, and appliances)
— the contribution of tap water used to water depletion (for housing, food, household goods, and appliances).

Finally, some substances are contributing to more than one impact category, and are then to be considered priority pollutants to be addressed. These are the emission of NOx to air (for acidification, marine eutrophication, terrestrial eutrophication, and, to some extent, photochemical ozone formation), the emission of NH_3_ to air (for acidification, terrestrial eutrophication, and particulate matter). Moreover, the emission of fossil CO_2_ to air (for climate change) and the emission of P to water and to soil (for freshwater eutrophication) are single substances, which alone cover a significant share of the related impact categories.

It is quite challenging to perform an external validation of the results because modelling assumptions (including system boundaries), the impact assessment method used and the approach applied (i.e., process-based LCA or input-output) may vary among studies. The impact on climate change (usually calculated with IPCC characterization factors) may, however, be a good indicator to use for comparison. Results of the present study indicate that household consumption in Europe generated a global warming potential of 9.55 t CO_2_eq/year per person. Results from other studies are quite diverse, also depending on the geographical area considered and the activities included. Kalbar et al. (2016) estimated a consumption-based global warming potential of 6.8 t CO_2_eq/year for Danish people, accounting for emissions related to accommodation, energy, road transport, air travel, and food. Kennedy et al. (2009) calculated total end-use emissions for the ten cities in the world, and they obtained values ranging from 4.2 t CO_2_eq/year to 21.5 t CO_2_eq/year. Within those cities, three of the four located in Europe showed values that are closer to the results of the present study (Geneva, 7.8 t CO_2_eq/year; Prague 9.4 t CO_2_eq/year: and London, 9.6 t CO_2_eq/year), whereas one corresponded to the lowest value of the range (Barcelona, 4.2 t CO_2_eq/year). Froemelt et al. (2018) developed a model to define archetypal behaviours of Swiss households and they built a hybrid LCA model to assess environmental impacts from household consumption and they found that the average household in Switzerland generates 9.0 t CO_2_eq/year per person.

Finally, two studies applying an input-output approach to assess the impact of UK households reported values that are close to the results of the present study: Baiocchi et al. (2010) report 8.3 t CO_2_eq/year, whereas Druckman and Jackson (2009) reported 9 t CO_2_eq/year. Eurostat (2011) reported 9 t CO_2_eq/year as well induced by the EU's final use of products. However, this includes also 1.6 t CO_2_eq/year due to investments (capital formation) in the EU economy.

### 3.3 Assessing changes of impacts of consumption over time

The analysis of apparent consumption in 2015 revealed a general increase in household consumption per person from 2010 to 2015, except some food products (milk, olive oil, potatoes, oranges, and tea) and some household goods (bar soap, shampoo, hair conditioner, furniture, and footwear). The most relevant increase is related to the number of kilometers travelled per person in one year (10% more in 2015 compared to 2010, affecting the impact of mobility) and the number of appliances owned (29% more in 2015 in total, with room air conditioner as the most relevant one in terms of increase, corresponding to 53%).

This increase is reflected in an increased overall impact generated by household consumption in the European Union. Because from 2010 to 2015 there was an extension of the geographical scope (from EU-27 to EU-28) and an increase in the population of around 1% (from 502 million people to 508 million people), the overall increase of impact per citizen is considered more informative of the evolution of the consumption patterns.

Hence, Fig. 2 shows the overall change in impacts per person, split into the contribution of the areas of consumption considered (i.e. the Bops), whereas figures in S5 (Figure S5.2, Figure S5.4, Figure S5.6, Figure S5.8 and Figure S5.10) show the change in impacts per person calculated for each BoP.

The impact per person from BoP Food increased from 7% to 17% from 2010 to 2015, depending on the impact category, because of the increase of the amount of food consumed by an average citizen (+6% in total, with significant increase for some products, such as cod +46% and farmed salmon, +45%) (Figure S5.6).

In the case of BoP Mobility, along with the increase of impacts in most of the impact categories (due to the increase in kilometres travelled per person - pkm), there are some reductions in 2015 compared to 2010. This reduction is due to the implication on particulate matter, photochemical ozone formation and land use, as a result of the introduction of new types of vehicles (electric and hybrid cars) and the reduction in the use of more impacting means of transport (e.g., diesel cars) (Figure S5.4).

BoP Housing is an exception to this trend because the impact generated in 2015 by the housing sector is lower than in 2010. The reduction (around 10% for most of the impact categories) is mainly driven by a general reduction of energy use in the buildings (especially for space heating), thanks to energy efficiency regulations in place since 2010 (Figure S5.2).

In the case of BoP Household goods, the impact generated by an average EU citizen in 2015 is higher than the one generated in 2010 for all the impact categories considered, due to the general increase in apparent consumption from 2010 to 2015. The increase of the impact per person is around 10% for most of the impact categories. One exception is the more limited increase of the impact on human toxicity cancer effect (5%), because of the lower contribution of footwear (and especially of fashion shoes, which in the BoP model are made by chrome-tanned leather). On the contrary, the impact on water use in 2015 is 20% higher than in 2010, because of the significant increase (55%) of toilet paper used. Since the use of water to flush the toilet is partially allocated to this product, the impact on water use arose consequently (Figure S5.10).

The impact generated by an average citizen with the purchase and use of appliances increased as well from 2010 to 2015. The increase is between 15% and 20% for almost all the impact categories. In the case of ozone depletion, the increase is higher (40% more in 2015 than in 2010), due to the significant increase (53%) in the number of air conditioners owned, and related use of refrigerants (Figure S5.8).

**Fig. 3:**
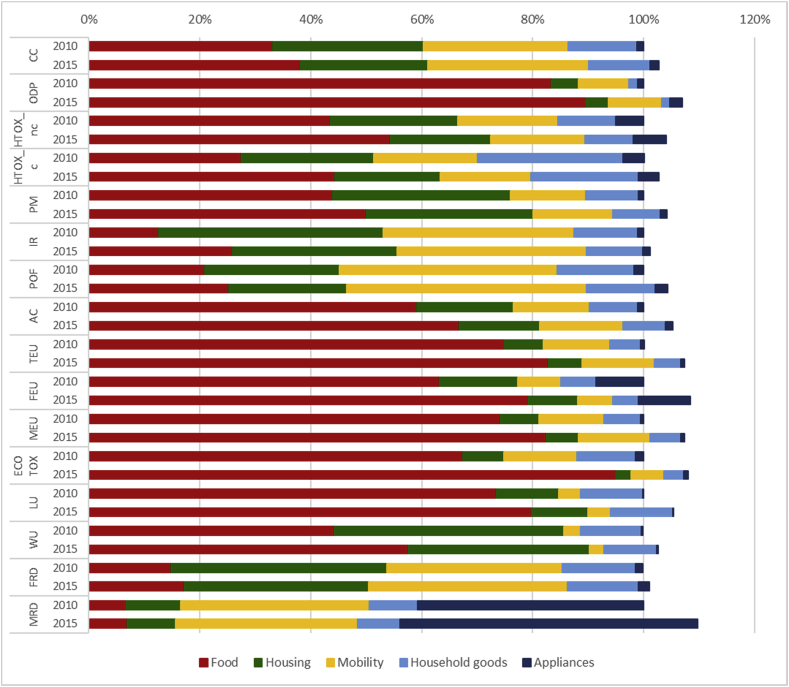
Comparison of Consumer Footprint in the reference year 2010 and in 2015 (with total impact of year 2010 set as 100%), illustrating the contributions of the different BoPs.

Also, the impact of total consumption (total BoPs) increased from 2010 to 2015 in all impact categories (Fig. 3). The highest increase is in the mineral and metal resource use (9%) due to the high increase in household appliances (see Fig. 3).

**Fig. 4:**
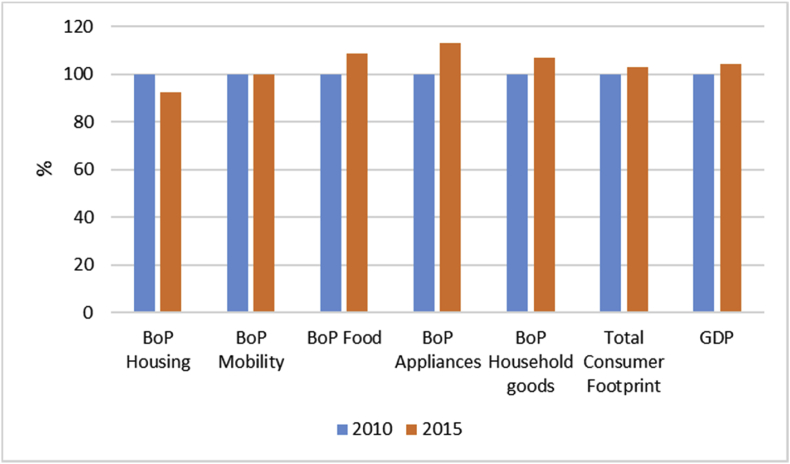
Percentage variation in the Consumer Footprint expressed as single score after weighting and the variation in GDP per person in year 2010 and in 2015.

When looking at weighted impacts of different BoPs per person, it emerges that the impact increases in all the areas of the consumption except housing (Fig. 4). The increase is higher for appliances, food, and household goods and more limited for mobility (+0.1%), due to the tradeoff between the increase in km and the improved efficiency of vehicles. The total Consumer Footprint per person (expressed as a single score after weighting) increased by 3% from 2010 to 2015, whereas the GDP per person increased by 4.5%. Therefore, it appears that there was a decoupling between GDP and impacts in the considered timeframe, even if quite limited.

### 3.4 Discussion of methodological aspects

The Consumer Footprint indicators allow assessing the environmental impacts induced by household consumption in the European Union, taking a life cycle-based perspective and using process-based LCA as methodology. Most of the previous studies calculated the impacts of consumption at European or World scale by using input-output approaches (Steen-Olsen et al., 2012; Ivanova et al., 2017; Tukker et al., 2016; Wood et al., 2018). Other studies applied process-based LCA or hybrid LCA focusing on one nation and a more limited set of products (e.g., Kalbar et al., 2016; Froemelt et al., 2018). However, to the Authors’ knowledge, this is the first study that applies process-based LCA to assess the impacts generated at the continental level (the European Union), and considering five areas of consumption. The method developed in this study implies some methodological challenges, and some limitations, which are discussed below.

#### 3.4.1 Identification and representativeness of the products

A crucial phase in the development of the BoP indicators is the initial identification of the selected basket categories (or consumption categories): food, mobility, housing, household goods, and appliances. The selection of the areas of consumption to be included has been done starting from the results of previous studies and considering the ones that were identified as more relevant, so to be able to cover most of the potential environmental impacts generated by consumption. Ideally, these categories should not overlap.

As the products included in the baskets were only a subset of total consumption, it has to be noted that the Consumer Footprint indicator was meant to be an index for monitoring and analysis, and not an absolute measure of environmental impact per person.

For example, the current selection still does not include 100% of the household activities - just a share resulting from the upscale, which is difficult to precisely determine-, and especially services. However, a study comparing bottom-up and top-down approaches, based on input-output tables, highlighted that services contribute to less than 10% of the impact generated by European households (Castellani et al., 2019b).

Complementing the study in the future could be interesting. For instance, to complete the impact profile of consumption by the average EU citizen, an extension with other less basic needs, e.g.: health-related sector (pharmaceutical and nutriceutical products, linked with new lifestyles and ageing of the population), and leisure activities including tourism (which is constantly showing increasing trends and market expansions). Those areas of consumption, despite being relatively smaller in economic terms compared to the others, may bring significant impacts (e.g., the relevance of climate impacts due to the tourism reported by Lenzen et al., 2018). After the definition of the five consumption categories (food, mobility, housing, household goods, appliances), the criteria used, and assumptions made to identify the representative products for each basket influence the estimation of the potential environmental impacts associated with the Consumer Footprint. On the one hand, the use of representative products leaves some product groups uncovered. For example, in the case of the BoP household goods, because of the large variety of product groups that may be included and because of the lack of inventory data for some product groups (e.g., pharmaceuticals). The impact generated by those product groups could be considered in the future by complementing the bottom-up approach followed for the BoP with information coming from top-down analysis (such as input-output assessment of household consumption).

On the other hand, the use of representative products may reduce the representativeness of the model, because it implies the modelling of products that represent the average products on the market. This could be a limitation especially for the stock of household appliances, which is composed by products with different levels of efficiency, also depending on their age. The same issue may become relevant when assessing the change of impacts over time, due to the more rapid evolution of some products (e.g., appliances) compared to other products that relatively more stable (e.g., food products), but which may in turn change in typology or main place of production.

Finally, allocation of public consumption and services to final consumption could be done as well. However, from previous study, this seems a relatively constant share of the overall consumption and it is barely dependent from individual behavior (which is what the consumer footprint aims to measure). Castellani et al., (2019b) and Beylot et al., (2019) did a comparison between top-down and bottom-up approaches, showing the relative importance of services, which account for around 10% of additional impacts.

#### 3.4.2 Model parametrization and suitability to run scenarios

Both the characteristics of the product and the composition of each BoP could consider evolutions over time. For example, improvement in production or in infrastructure might be reflected in less emission per products at the production stage, as well as a different composition of the basket may take into account different consumers choices and behaviors (either in the choice of products or in their use, especially when it implies energy use). The process-based LCA allow this flexibility, being able to model consumer behavior and the effect of consumer behaviors’ drivers. An overview on the potential contribution of behavioral science to LCA is presented in a dedicated report (Nita et al., 2017), and Polizzi di Sorrentino et al., 2016 has depicted a preliminary methodological framework for coupling consumer behaviour and LCA.

#### 3.4.3 Defining different baskets based on product performances

Towards further development of the Consumer Footprint indicators, it seems relevant to define and account for not only single baskets for each category (e.g. one set of representative products for the category food), but – ideally – a range of different baskets from the “best performing” to the “worst performing” one from an environmental point of view. An additional option in this direction is the development of country-specific baskets, i.e. baskets that include country-specific representative products, to take into account also the specific purchasing behaviour of the consumers and the differences in the trade (e.g. differences in the countries from which products are imported, or differences in share of imported goods). The feasibility of this option should, of course, be evaluated via, e.g. ad-hoc studies aimed at identifying country-specific representative products as well as the availability of the necessary country-specific data (of sufficient quality) to conduct the calculations. While in the short-term the development of entirely country-specific baskets may not be straightforward, a progressive adaptation of the baskets to include national specificities seems viable. The feasibility of using input-output data to complement the LCI data used so far can be considered to support this purpose, as these data provide more extensive country-specific information, as well as, in some cases, relevant life cycle stage-specific information.

In general, increased availability of high-quality LCI data would help increasing comprehensiveness and robustness of the Consumer Footprint indicators. In order to limit data collection efforts (and costs), the calculations of Consumer Footprint indicators so far conducted should be carefully checked (via, e.g. sensitivity analysis) to identify key hotspots, product groups, lifecycle stages, etc. that mostly influence the environmental performance. Data collection efforts could then be limited to these identified components, at least in the short term.

As a general consideration, the bottom up-methods hold a realistic picture and a high level of detail at the level of specific products, but they are not designed so to be consistent with national or sectorial statistics reporting total emissions. In addition to that, this group of methods commonly neglects indirect economic effects due to prices being considered as exogenous. Hence, any assessment of the rebound effect (Vivanco et al., 2018) should come from complementing the bottom-up approach with other modelling approaches.

### 3.5 Outlook on the potential use of the Consumer Footprint for the SDG 12 and for policy support

The Consumer Footprint results and the assessment of their evolution over time might be relevant for supporting policies in many different areas. First of all, for sustainable production and consumption policies (e.g. starting from the EU Sustainable Consumption and Production Action Plan; EC, 2008 up to the SDG 12 on responsible consumption and production), the development of consumption-based indicators are quite crucial for highlighting and prioritising hotspots of impacts due to consumption in terms of: product groups, consumption patterns, key life cycle stages driving the impacts. Moreover, the use of a life cycle assessment approach to support policies is advocated by the EU in the context of the Better regulation toolbox (EC, 2015a). Among the others, the Consumer Footprint may support eco-innovation related policies (e.g., according to the EU eco-innovation action plan; EC, 2011b) and circular economy policies (EC, 2015b).

The life cycle indicators framework and the resulting indicators can be used to address a variety of policies, such as the circular economy, the bioeconomy and related policies, e.g., industrial policy. Based on the information provided by life cycle indicators, policy scenarios can be analysed (e.g. in the context of the impact assessment of policy options), evaluating the trade-offs associated with policies and guiding their developments. To assess the environmental impact of the final consumption the Consumer Footprint is extremely relevant.

Since 2011, policies such as the Resource-efficient Europe flaghship initiative (EC, 2011a) explicitly mention the need for indicators that measure environmental impacts and our natural capital or ecosystems as well as take into account the global aspects of EU consumption.

Moreover, policy targets could be based on the use of LCA. As general definition, targets are a quantitative measure of reduction (or increase) from a given reference, regardless of the scale (macro, meso, micro), foreseen over time (Sala et al., 2014; Moldan et al., 2012). LCA results, such those of the Consumer Footprint, may represent benchmark against which to set reduction targets (e.g. reduction of consumption-based carbon footprint from the 9.55 t CO_2_eq/year per person of an average EU citizen to the 1 t CO_2_eq/year as advocated by initiative such as the "1 ton society", Climate-KIC 2019).

Moreover, the added value of the Consumer Footprint would be the possibility of: i) optimizing different policy objectives, as 16 different environmental impact categories are covered in the assessment, and ii) better unveiling trade-offs related to transition towards less impacting lifestyle such, e.g., a more efficient technology may have a lower impact in the use phase while being more impacting in the production or EoL.

Regarding targets on specific environmental aspects (e.g., emissions, resource consumption, recyclability, waste production, etc.), the Consumer Footprint may support the analysis of hotspots and the definition of impact reduction measures.

Indeed, a life cycle-based indicator as the Consumer Footprint may also help the assessment of future consumption scenarios, because the framework is flexible and several assumptions could be made on future policy-driven, behaviour-driven or technologically-driven scenarios. Alternative scenarios, e.g. related to a different composition of a basket in an evolving consumption system could be assessed. For example, the consumption in future of less meat and more vegetal protein may change the composition of the basket and the related figures for the 16 impact categories, highlighting if and where an environmental benefit or burden-shifting takes place. Conversely, assuming a relative stable composition of the basket, different technological options as results of policy interventions could be assessed in terms of relative benefits (e.g. if increasing the energy efficiency of a specific typology of houses is leading to a tangible benefit in climate-change related impact or also in other impact categories considering the whole life cycle).

All these aspects enable Consumer Footprint to be considered suitable for both monitoring the SDG12 and evaluate options to reach the different impact reduction targets. In fact, the Consumer Footprint could be used to monitor sustainable production and consumption over time, with a supply chain perspective, to identify hotspots as priority areas of consumption and products on which to focus policies. Moreover, taking the Consumer Footprint in a reference year as baseline, it is possible to test actions toward more sustainable production and consumption patterns.

Moreover, Consumer Footprint calculations may support the assessment of current lifestyles and the effort needed, e.g. towards climate-neutral ones. As mentioned above project such as the one promoting a “1 ton society” (Climate-KIC, 2019) is advocating lifestyle which emissions per capita and per year are 1 ton of CO_2_. In 2010, the Consumer footprint for climate change in EU was of 9.5 ton of CO_2_eq per person per year. This is giving a clear indication on the magnitude of reduction needed and how different areas of consumption and choices are affecting the overall impacts.

Finally, addressing SDG 12 with the Consumer Footprint could be seen not only in relation to environmentally-relevant SDGs but broadly related to human health implications as well (e.g. SDG3). Studies addressing the interplay between climate neutral lifestyles and human health are emerging (see e.g. Quam et al., 2017) and Consumer Footprint might be pivotal in assessing benefits and trade-offs.

## 4 Conclusions

The present study aimed at assessing the environmental impact of EU consumption using process-based LCA of representative products. The assessment of the Consumer Footprint in the European Union highlighted that consumption of food is the primary driver of impacts for most of the impact categories considered, followed by housing and mobility (especially for climate change, photochemical ozone formation, fossil resource use and mineral, and metal resource use). Appliances are the main driver for the impact category mineral and metal resource depletion. Household goods are generally less relevant than the other areas of consumption considered. However, modelling efforts are needed to ensure better coverage of the consumed household goods, which may ultimaltely imply an increase of their relativel relevance.

The analysis conducted so far on the five BoPs that compose the Consumer Footprint indicator allows for identifying which are the products driving the impact in those areas of consumption. Dwellings in the moderate climate have the highest contribution to the environmental impacts of housing due to the highest number of dwellings in that area. The use phase contributes more than 50% to the overall impact of the dwellings, for most of the impact categories. Passenger cars are the most contributing mode of transport among the transportation used by European citizens. The second highest contributor is air transport. The food product groups that emerge as hotspots in most of the impact categories are meat, dairy products, and beverages. Main contributing processes are related to animal feeding. The most contributing household appliances are dishwasher, washing machine, refrigerator, lighting, and TV screen, with different shares depending on the impact category considered. The most contributing household goods are paper products, detergents, furniture, and clothes, again with different shares depending on the impact category considered.

Across the BoPs, the use phase is the most important life cycle stage for many impact categories, especially for the areas of consumption housing, mobility, and appliances. For the BoP Food, the most important life cycle phase is upstream, which corresponds to agricultural activities. In the case of mobility, the production phase appears to be particularly relevant, because it includes both the manufacture of the means of transport and the production of their components.

Apart from agriculture, which is the main hotspot in the food life cycle, the main hotspots identified in terms of activities are heating of buildings, burning of fossil fuels in the use of passenger cars, and especially diesel ones, and electricity use (for heating, cooling, lighting, and the use of household appliances).

When looking at the change in the impacts generated in 2010 and 2015, a general increase is observed. In the case of BoP food, BoP appliances and BoP household goods, the environmental impacts generated in 2015 are higher compared to 2010, due to a general increase in apparent consumption. In the case of BoP mobility, the environmental impact is generally higher due to an increase in km travelled. On the contrary, impacts generated in 2015 by the housing sector were lower than in 2010 due to an increase of the energy efficiency of the building stock.

The presented work follows the current state-of-the-art in estimating the impact of final consumption. Although further methodological improvements are needed, the results depict a fair quantification of the impact, highlighting specific hotspots within the life cycle stages of the representative products. Hence, the approach could be used to monitor the evolution of the impacts due to production and consumption overtime. Indeed, results in a reference year may be used as the baseline for monitoring purposes or as the benchmark against which to test scenarios related to products with different performance, different consumer behaviours, implementation of new infrastructure etc. Potential policy measures should be translated into scenarios with specific changes in the BoPs, where the methodology presented here should allow a user to forecast the changes in environmental impact for the consumption category as such, but also the change for the final consumption as a whole.

Finally, results could be useful as comprehensive indicators for monitoring the SDG12. The Consumer Footprint indicators encompass all life cycle stages of production and consumption and, if adequately updated, may help to assess evolution of impacts - over time- in response to changes in consumption patterns.
