# Supplementary material for: The consumer footprint: Monitoring sustainable development goal 12 with process-based life cycle assessment
Source: J Clean Prod. 2019 Dec 10;240:118050. doi: 10.1016/j.jclepro.2019.118050 (PMC6886560; doi:10.1016/j.jclepro.2019.118050)
Supplement: Multimedia component 1 [file mmc1.docx]

**The Consumer Footprint: addressing Sustainable Development Goal 12 with process-based life cycle assessment**

Serenella Sala, Valentina Castellani

^a^European Commission-Joint Research Centre, Via E Fermi 2749, I-21027 Ispra (VA), Italy

**SUPPLEMENTARY MATERIAL**

Contents

[S1 Environmental footprint impact assessment method, global normalization factors, and weighting factors 2](#_Toc523233899)

[S2 Modelling of BoPs in 2010 and 2015 – representative products and quantities 4](#_Toc523233900)

[S3 Overlaps of activities and actions taken to avoid double counting in the sum of the BoPs 11](#_Toc523233901)

[S4 Environmental impacts per person 13](#_Toc523233902)

[S5 Impacts of single baskets in 2010 and 2015 14](#_Toc523233903)

[References 22](#_Toc523233904)

## S1 Environmental footprint impact assessment method, global normalization factors, and weighting factors

Table S1.1-Impact category, underpinning models, units, robustness of the impact assessment model as defined in the Guidance for the development of Product Environmental Footprint Category Rules (PEFCRs) (EC, 2017)

| **Impact category** | **Abbreviation** | **Unit** | **Model** | **Robustness** |
| --- | --- | --- | --- | --- |
| Climate change | CC | kg CO_2_ eq | IPCC, 2013 | I |
| Ozone depletion | ODP | kg CFC-11 eq | World Meteorological Organisation (WMO), 2014 | I |
| Human toxicity, non-cancer | HTOX_nc | CTUh | USEtox (Rosenbaum et al., 2008) | II/III |
| Human toxicity, cancer | HTOX_c | CTUh | USEtox (Rosenbaum et al., 2008) | II/III |
| Particulate matter | PM | Disease incidence | Fantke et al., 2016 | I |
| Ionising radiation | IR | kBq U-235 eq. | Frischknecht et al., 2000 | II |
| Photochemical ozone formation | POF | kg NMVOC eq. | Van Zelm et al., 2008, as applied in ReCiPe 2008 | II |
| Acidification | AC | mol H^+^ eq | Posch et al., 2008 | II |
| Eutrophication, terrestrial | TEU | mol N eq | Posch et al., 2008 | II |
| Eutrophication, freshwater | FEU | kg P eq | Struijs et al., 2009 | II |
| Eutrophication, marine | MEU | kg N eq | Struijs et al., 2009 | II |
| Land use | LU | Pt | De Laurentiis et al. 2019, based on Bos et al., 2016 | III |
| Ecotoxicity freshwater | ECOTOX | CTUe | USEtox (Rosenbaum et al., 2008) | II/III |
| Water use | WU | m^3^ water eq of deprived water | AWARE 100 (based on) (UNEP 2016; Boulay et al. 2018a) | III |
| Resource use, fossils | FRD | MJ | ADP fossils (van Oers et al., 2002) | III |
| Resource use, minerals and metals | MRD | kg Sb eq | ADP ultimate reserve (van Oers et al., 2002) | III |

Table S1.2 Global normalization factors and weighting factors used for the Environmental Footprint

| **Impact category** | **Abbreviation** | **Unit** | **Global normalization factor^a^** | **Global normalization factor per person^a^** | **Weighting factor^b^** |
| --- | --- | --- | --- | --- | --- |
| Climate change | CC | kg CO_2_ eq | 5.55E+13 | 8.04E+03 | 21.06 |
| Ozone depletion | ODP | kg CFC-11 eq | 3.33E+08 | 4.84E-02 | 6.31 |
| Human toxicity, non-cancer | HTOX_nc | CTUh | 2.66E+05 | 3.85E-05 | 2.13 |
| Human toxicity, cancer | HTOX_c | CTUh | 3.27E+06 | 4.75E-04 | 1.84 |
| Particulate matter | PM | Disease incidence | 4.11E+06 | 5.95E-04 | 8.96 |
| Ionising radiation | IR | kBq U-235 eq. | 9.54E+11 | 1.38E+02 | 5.01 |
| Photochemical ozone formation | POF | kg NMVOCeq. | 2.80E+11 | 4.06E+01 | 4.78 |
| Acidification | AC | mol H^+^ eq | 3.83E+11 | 5.55E+01 | 6.20 |
| Eutrophication, terrestrial | TEU | mol N eq | 1.22E+12 | 1.77E+02 | 3.71 |
| Eutrophication, freshwater | FEU | kg P eq | 1.11E+10 | 1.61E+00 | 2.80 |
| Eutrophication, marine | MEU | kg N eq | 1.35E+11 | 1.95E+01 | 2.96 |
| Land use | LU | Pt | 1.54E+16 | 2.23E+06 | 7.94 |
| Ecotoxicity freshwater | ECOTOX | CTUe | 8.15E+13 | 1.18E+04 | 1.92 |
| Water use | WU | m^3^ water eq | 7.91E+13 | 1.15E+04 | 8.51 |
| Resource use, fossils | FRD | MJ | 4.48E+14 | 6.53E+04 | 8.32 |
| Resource use, minerals and metals | MRD | kg Sb eq | 4.39E+08 | 6.36E-02 | 7.55 |

^a^ Source: modified from Sala et al. (2017). Figures in blue refers to updated normalisation factors compared to those in the source.

^b^ Source: Sala et al. (2018b)

## S2 Modelling of BoPs in 2010 and 2015 – representative products and quantities

The following sections illustrate the composition of each BoP (in terms of representative products and quantities) and the variation occurred between 2010 and 2015.

**BoP Housing**

The basket is composed by 24 reference dwellings, representative of the EU-27 housing stock in the year 2010, divided by type of building, climate zone and year of construction. The features chosen to define the representative dwellings in the BoP-housing are:

- the dwelling type: Multi-family house (MFH) or Single- Family House (SFH)
- the climate of the area in which the building is located: cold, moderate or warm
- the period of construction: before 1945, between 1946 and 1969, between 1970 and 1989, between 1990 and 2010.

The model of BoP Housing has been updated to represent the EU building stock in 2015, by mean of two main modifications. Firstly, new dwellings in buildings built from 2010 to 2015 have been added to the existing stock (Table S2.1). Secondly, each of the dwelling archetypes has been updated to represent the average dwelling features in 2015. The features that have been updated are the average dwelling area, the number of people leaving in the dwelling and the energy used for space heating and the use of electric appliances.

*Table S2.1. Number of dwellings by dwelling archetype in the model of the BoP Housing in 2010 and in 2015*

| **Building type** | **Climate area** | **Year of construction** | **Total number of dwellings**  **(2010)** | **Total number of dwellings**  **(2015)** | **Variation** |
| --- | --- | --- | --- | --- | --- |
| **SFH** | WARM | <1945 | 3,990,078 | 3,553,315 | -11% |
|  |  | 1945-1969 | 3,940,268 | 3,603,680 | -9% |
|  |  | 1970-1989 | 5,029,842 | 4,871,429 | -3% |
|  |  | 1990-2010 | 3,015,954 | 3,848,946 | 28% |
|  |  | 2011-2015 | - | 685,347 |  |
|  | MODERATE | <1945 | 19,053,376 | 23,240,475 | 22% |
|  |  | 1945-1969 | 21,741,474 | 22,498,384 | 3% |
|  |  | 1970-1989 | 24,874,549 | 22,411,996 | -10% |
|  |  | 1990-2010 | 15,835,402 | 14,315,813 | -10% |
|  |  | 2011-2015 | - | 3,072,409 |  |
|  | COLD | <1945 | 1,137,005 | 960,326 | -16% |
|  |  | 1945-1969 | 1,123,212 | 1,237,656 | 10% |
|  |  | 1970-1989 | 1,258,137 | 1,307,100 | 4% |
|  |  | 1990-2010 | 629,666 | 595,889 | -5% |
|  |  | 2011-2015 | - | 110,534 |  |
| **MFH** | WARM | <1945 | 5,563,385 | 4,954,404 | -11% |
|  |  | 1945-1969 | 10,977,814 | 10,040,060 | -9% |
|  |  | 1970-1989 | 12,326,198 | 11,937,990 | -3% |
|  |  | 1990-2010 | 6,923,950 | 8,836,311 | 28% |
|  |  | 2011-2015 | - | 1,573,401 |  |
|  | MODERATE | <1945 | 12,883,862 | 15,715,171 | 22% |
|  |  | 1945-1969 | 16,543,072 | 17,119,005 | 3% |
|  |  | 1970-1989 | 19,849,947 | 17,884,824 | -10% |
|  |  | 1990-2010 | 11,961,082 | 10,813,279 | -10% |
|  |  | 2011-2015 | - | 2,320,708 |  |
|  | COLD | <1945 | 1,326,949 | 1,120,755 | -16% |
|  |  | 1945-1969 | 1,580,981 | 1,742,067 | 10% |
|  |  | 1970-1989 | 1,831,828 | 1,903,117 | 4% |
|  |  | 1990-2010 | 910,548 | 861,704 | -5% |
|  |  | 2011-2015 | - | 159,841 |  |
| *Total* | | | *204,479,230* | *213,295,936* | *5%* |

As shown in Table S2.1, the number of dwellings considered in BoP Housing for 2015 is 5% higher than the one in 2010 model (also because of the shift from EU-27 to EU-28). On the contrary, the total amount of energy used in this larger number of dwellings is 4% less than it was in 2010 (Table S2.2). In addition, there has been a reduction in the amount of energy from fossil sources (coal, oil and gas) and of the electricity taken form the grid, combined with an increase in the amount of energy produced from renewable source and of heat from waste incineration.

*Table S2.2. Amount of dwellings and total energy use for housing in Europe in 2010 and 2015*

|  | **2010** | **2015** | **Variation** |
| --- | --- | --- | --- |
| Dwellings | 204,479,230 | 213,295,936 | 5% |
| Total energy use for housing in Europe (kWh/y) | 3.57*10^12^ | 3.43*10^12^ | -4% |
| Total energy use in for housing Europe by energy source (kWh/y) | | | |
| Coal | 1.27E+11 | 1.19E+11 | -7% |
| Oil | 4.71E+11 | 4.43E+11 | -6% |
| Gas | 1.33E+12 | 1.28E+12 | -4% |
| Heat from incineration | 2.48E+11 | 2.65E+11 | 7% |
| Renewable sources | 5.02E+11 | 5.14E+11 | 2% |
| Electricity from grid | 8.90E+11 | 8.10E+11 | -9% |

When analysing those data, it has to be considered that some of the data sources used to model BoP Housing for the year 2015 differ from the ones used to model BoP Housing for 2010. The main source used for 2015 model is the EU Buildings database^^[[1]](#footnote-1)^^, integrated with some data from Eurostat. The IEE Projects ENTRANZE (2014)^[[2]](#footnote-2)^, TABULA (2012)^[[3]](#footnote-3)^ and EPISCOPE (2012)^[[4]](#footnote-4)^, which were used to model BoP Housing for 2010 year, were not updated further, so they could not be used for 2015. Therefore, it is difficult to understand to which extent the observed change is due to a real reduction of the energy used in European buildings, and how much is due to inconsistencies between the sources used. However, the trend shown by data reported in Table S2.2 is confirmed also by other studies (e.g. ODYSSEE-MURE, 2015)^[[5]](#footnote-5)^. The main reason for the reduction of the total energy used is the implementation of energy efficiency regulations in existing buildings and the higher energy efficiency of new buildings, built after 2010.

**BoP Mobility**

A selection of 27 representative means of transport was made to represent the fleet composition in Europe in 2010 in the BoP Mobility, as follows:

- 9 types of Gasoline passenger cars (with 3 engine capacities: <1.4L, between 1.4-2.0L and >2.0L, and 3 emissions standards: Euro 0-Euro 3, Euro 4 and Euro 5)
- 6 types of Diesel passenger cars (with 2 engine capacities: between 1.4-2.0L and >2.0L, and 3 emissions standards: Euro 0-Euro 3, Euro 4 and Euro 5)
- LPG passenger car
- 3 types of 2-wheelers (mopeds, motorcycles <250cc, motorcycles >250cc)
- 3 types of buses (diesel urban buses, CNG urban buses and coaches)
- 2 types of trains (electric and diesel)
- 3 types of flights (national, intra-EU and extra-EU)

Passenger mobility in Europe showed an increase (+10%) of km travelled by European citizens from 2010 to 2015 (also due to the extension of the geography, form EU-27 to EU-28). Regarding the means of transport used, the highest variation happened for public transport, and especially for urban buses and coaches. The use of trains and air transport increased as well (Table S2.3).

*Table S2.3. Means of transport in the model of BoP Mobility and kilometres travelled in Europe in 2010 and 2015*

| **Mean of transport** | | **Passenger kilometres (pkm)** | | **Variation** |
| --- | --- | --- | --- | --- |
|  |  | **2010** | **2015** |  |
| Passenger cars | Gasoline | 4.71E+12 | 4.87E+12 | 3% |
|  | Diesel |  |  |  |
|  | LPG |  |  |  |
|  | Electrical |  |  |  |
|  | Hybrid |  |  |  |
| 2W | | 1.40E+11 | 1.30E+11 | -7% |
| Urban buses and Coaches | | 4.18E+11 | 5.46E+11 | 27% |
| Trains | | 4.04E+11 | 4.90E+11 | 21% |
| Aircrafts | | 2.72E+12 | 3.22E+12 | 18% |
| *Total* | | *8.39E+12* | *9.26E+12* | *10%* |

**BoP Food**

The BoP Food consists of a basket of the most relevant food product groups, selected by importance in mass and economic value, to depict the average consumption for nutrition of EU citizens in 2010. The BoP Food also includes products that are representative of emerging food consumption trends and types of food and beverages whose consumption has been increasing during the past decade (e.g. tofu, pre-prepared meals, etc.).

The variation in the consumption of food products per person from 2010 to 2015 is 6% in total, with significant increase in the consumption of some products (e.g. 46% for salmon and cod) and significant reduction for other products (e.g. -34% for oranges and -33% for tea) (Table S2.4).

*Table S2.4. Amount of products (per person) in the model of BoP Food in 2010 and 2015*

| **Product Group** | **Representative product** | **Per-capita consumption (kg/pers.*yr^-1^)** | | **Variation** |
| --- | --- | --- | --- | --- |
|  |  | **2010** | **2015** |  |
| MEAT | Pork meat | 41 | 44.9 | *8.7%* |
|  | Beef meat | 13.7 | 15.2 | *9.9%* |
|  | Poultry meat | 22 | 26.3 | *16.3%* |
| SEAFOOD | Farmed salmon | 1.9 | 3.5 | *45.7%* |
|  | Cod (wild) | 5.6 | 10.4 | *46.2%* |
|  | Shrimps | 1.4 | 1.5 | *6.7%* |
| DAIRY | Milk | 79.7 | 78.4 | *-1.7%* |
|  | Cheese | 15 | 15.1 | *0.7%* |
|  | Butter | 3.6 | 4.4 | *18.2%* |
| EGGS | Eggs | 13.2 | 14 | *5.7%* |
| CEREAL-BASED PRODUCTS | Bread | 39.3 | 40 | *1.8%* |
|  | Pasta | 8.2 | 9.3 | *11.8%* |
|  | Rice | 9 | 9.6 | *6.3%* |
| SUGAR | Sugar | 27.8 | 28.6 | *2.8%* |
| OILS | Sunflower oil | 5.3 | 5.7 | *7.0%* |
|  | Olive oil | 5.3 | 4.7 | *-12.8%* |
| TUBERS | Potatoes ^a^ | 69.1 | 68.5 | *-0.9%* |
| VEGETABLES | Tomatoes | 12.8 | 14.5 | *11.7%* |
| LEGUMES | Beans | 2.3 | 2.8 | *17.9%* |
|  | Tofu ^b^ | 4.3 | 5.1 | *0.2%* |
| FRUITS | Oranges | 17.4 | 13 | *-33.8%* |
|  | Apples | 16.1 | 17.5 | *8.0%* |
|  | Bananas | 10.3 | 11.5 | *10.4%* |
| NUTS | Almonds ^a^ | 0.6 | 0.6 | *0.0%* |
| COFFEE & TEA | Coffee | 3.5 | 3.3 | *-6.1%* |
|  | Tea | 0.8 | 0.6 | *-33.3%* |
| BEVERAGES | Beer | 69.8 | 70 | *0.3%* |
|  | Wine | 24 | 26 | *7.7%* |
|  | Mineral water | 105 | 122.3 | *14.1%* |
| CONFECTIONERY PRODUCTS | Biscuits | 6.9 | 7.1 | *2.8%* |
|  | Chocolate | 4.1 | 6 | *31.7%* |
| PRE-PREPARED MEALS | Meat based dishes | 2.9 | 3.4 | *14.7%* |
| *Total* | | 642 | 683 | *6%* |

^a^ The amount for year 2015 was not available; amount in 2015 is based on 2013 data

^b^ Amount of tofu is based on EFSA (2018), because data on apparent consumption was not available. EFSA data is from national dietary surveys, which have been carried out in different years in different countries. However, available data for years 2010 and 2015 was so limited (only couple of countries), that all data was used to calculate average, which was used as reference amount in 2010. However, consumption of vegetarian food, as well as soy-based food, is constantly increasing (Askew, 2017). In addition, soy cultivation is constantly increasing, which is partly due to increased use of soy as animal feed and biofuels, but also increased use in food products (WWF, 2018). Data of increase in consumption of soy-based food was not available, so the same increase with beans have been used, because both are vegetarian options to replace animal protein intake in diet.

**BoP Appliances**

The BoP Appliances consist of a process-based LCI model for a basket of products that represent the most relevant household appliances in terms of energy consumption and market share in Europe. The amount of representative products included in the BoP Appliances is calculated starting from the analyses of the existing stock done for the Ecodesign preparatory studies. For each representative product, the whole stock present in European households is allocated to the reference year (dividing it by the number of service life years of the representative product chosen), and then to the number of users (i.e. European citizens in the reference year). This results in a per-capita yearly consumption expressed in pieces/person*year^-1^.

Similarly to what happens for the other BoPs, the BoP Appliances shows an increase in the amount of devices owned and used by an average European citizen in 2015 compared to 2010. In addition, LED lighting has been introduced in 2015, to take into account the phasing-out of incandescent lamps and the progressive substitution of halogen ones (Table S2.5).

*Table S2.5. Amount of products in BoP Appliances in 2010 and 2015*

| **Representative Product** | **Total stock of the product group in 2010 (pieces)** | **Total stock of the product group in 2015 (pieces)** | **Amount per capita 2010 (pieces/pers*y^-1^)** | **Amount per capita 2015 (pieces/pers*y^-1^)** | **Variation of amount per capita (%)** |
| --- | --- | --- | --- | --- | --- |
| Dishwasher 10 ps | 1.24E+07 | 1.48E+07 | 0.002 | 0.002 | 15% |
| Dishwasher 13 ps | 7.04E+07 | 8.40E+07 | 0.011 | 0.013 | 20% |
| Washing Machine | 1.86E+07 | 1.98E+08 | 0.030 | 0.031 | 4% |
| Tumble dryer | 6.30E+07 | 6.84E+07 | 0.006 | 0.006 | 2% |
| Combined refrigerators-freezers | 2.99E+08 | 3.05E+08 | 0.022 | 0.022 | 2% |
| Air conditioner, single split | 2.81E+07 | 4.65E+07 | 0.004 | 0.006 | 53% |
| Electric oven (built-in) | 2.16E+08 | 2.21E+08 | 0.010 | 0.011 | 8% |
| Compact fluorescent lamp | 1.49E+09 | 1.71E+09 | 0.246 | 0.280 | 14% |
| Halogen lamp, low voltage | 9.03E+08 | 8.54E+08 | 0.408 | 0.382 | -6% |
| Halogen lamp, mains voltage | 1.06E+09 | 8.54E+08 | 0.638 | 0.509 | -20% |
| Incandescent lamp | 7.16E+08 | 1.51E+09 | 0.648 | 1.350 | 108% |
| LED | - | 1.00E+08 | - | 0.006 | 100% |
| Notebook | 1.79E+08 | 2.31E+08 | 0.028 | 0.036 | 30% |
| LCD TV screen | 3.32E+08 | 4.08E+08 | 0.058 | 0.071 | 22% |
| *Total* | *5.39E+09* | *6.60E+09* | *2.11* | *2.73* | *29%* |

**BoP Household goods**

The BoP Household goods consists of a process-based LCI model for a basket of products that represent the most relevant product groups consumed in households. The selection of the product groups to be included in the basket was based mainly on the list of product groups already covered by the Ecolabel and for which Green Public Procurement (GPP) criteria were available, complemented with the product groups for which a Product Environmental Footprint (PEF) pilot was ongoing. The reason of this choice is that the selection of product groups that are covered by Ecolabel or GPP criteria follows a set of criteria (including market significance in terms of stock volume and sales and importance of the environmental impact generated) that is in line with the ones that drove the selection of the representative products for the other BoPs.

The change in apparent consumption of household goods between 2010 and 2015 is more variable than for other BoPs, with some goods (as liquid soap) showing an increase higher than 100% and some others (e.g. hair conditioner) showing a reduction close to 40% (Table S2.6). This variation should be interpreted with caution, because of the limited number of representative products included in the basket and the upscale of quantities.

*Table S2.6 Amount of products in BoP Household goods in 2010 and 2015*

| **Product Group** | **Representative product** | **Unit** | **Per capita apparent consumption upscaled (unit/pers*y^-1^)** | | |
| --- | --- | --- | --- | --- | --- |
|  |  |  | **2010** | **2015** | **Variation** |
| Liquid and powder detergents | All-Purpose Cleaners and Sanitary Cleaners | kg | 9.99 | 11.32 | 13% |
|  | Detergents for Dishwashers | kg | 2.43 | 2.75 | 13% |
|  | Hand Dishwashing Detergents | kg | 1.75 | 1.98 | 13% |
|  | Laundry Detergents liquid | kg | 10.03 | 11.36 | 13% |
|  | Laundry Detergents powder | kg | 3.10 | 3.51 | 13% |
| Absorbent Hygiene Products | Baby diapers | kg | 3.22 | 5.24 | 63% |
|  | Sanitary pads | kg | 4.05 | 6.44 | 59% |
|  | Tampons | kg | 0.09 | 0.14 | 54% |
|  | Breast pads | kg | 0.56 | 0.91 | 63% |
| Rinse-off Cosmetic Products (Soaps and shampoos) | Bar soap | kg | 4.59 | 4.06 | -12% |
|  | Liquid soap | kg | 1.82 | 4.18 | 130% |
|  | Shampoo | kg | 3.13 | 2.04 | -35% |
|  | Hair conditioner | kg | 2.07 | 1.26 | -39% |
| Furniture | Bedroom wooden furniture | pieces | 0.20 | 0.19 | -2% |
|  | Kitchen furniture | pieces | 0.30 | 0.27 | -10% |
|  | Upholstered seat | pieces | 0.19 | 0.15 | -19% |
|  | Non-Upholstered seat (wooden seat) | pieces | 0.27 | 0.18 | -35% |
|  | Wooden table | pieces | 0.17 | 0.18 | 10% |
| Bed Mattresses | 3 types: Latex, PUR and spring | pieces | 0.09 | 0.10 | 10% |
| Footwear | WW | pairs | 0.48 | 0.45 | -7% |
|  | Sport | pairs | 0.61 | 0.82 | 35% |
|  | Leisure | pairs | 2.29 | 2.13 | -7% |
|  | Fashion | pairs | 2.29 | 2.13 | -7% |
| Textile products | T-shirt | pieces | 31.80 | 34.70 | 9% |
|  | Women blouse | pieces | 8.55 | 9.32 | 9% |
|  | Men trousers | pieces | 3.74 | 4.08 | 9% |
|  | Jeans ^a^ | pieces | 4.79 | 5.22 | 9% |
| Paper products | Newsprint | kg | 90.50 | 85.05 | -6% |
|  | Book | kg | 19.26 | 25.07 | 30% |
|  | Toilet paper | kg | 23.55 | 36.39 | 55% |

^a^ The amount of textile in 2015 and, consequently, its increase with respect to 2010 data, is based on the economic value increase of the whole textile sector due to a low robustness of considered data.

## S3 Overlaps of activities between different BoPs and actions taken to avoid double counting in the sum of the BoPs

Table S3.1. Summary of the possible overlaps among the baskets, and related choice on where to keep the activity and rationale of the choice.

| **Life Cycle Stages** | **Activity** | **Comment** | **Basket in which is kept** | **Rationale for the choice** |
| --- | --- | --- | --- | --- |
| *Use* | transport to client | Transport to client overlaps among the use phase of the Food, Mobility, Household goods, and lighting of the Appliances basket. | Mobility | Since BoP Mobility is more comprehensive as it includes the overall use of passenger car, the activity is kept in that basket, to have the overall footprint of transport in the Mobility sector. |
|  | tap water | Tap water use is overlapped among Food, Housing, and Household goods basket. | Housing | European average value of 150 litres per person per day was assumed in case of Housing. Cooking of Food is included here. The top-down statistics for Housing is considered tidier than the bottom-up numbers in case of Food, Household goods, and Appliances. |
|  | heat | Heat consumption overlaps with the use phase of Food and Housing basket. | Housing | Energy consumption data in the Housing basket are from the EU-27 energy statistics and valid for the total dwelling stock. As a general rule, energy consumption in the use phase is kept in the BoP housing, which is more comprehensive compared to the others. |
|  | electricity | Electricity consumption overlaps with the use phase of Food, Housing, Household goods, and Appliances basket. | Housing | Because energy consumption data in the Housing basket are from the EU-27 energy statistics which is more comprehensive. |
|  | detergents | Use of detergents overlaps between the basket Appliances and Household goods. | Household goods | It makes more sense to keep the amount of detergents used in the use phase of the product detergents than in the use phase of the appliances. |
| *Wastewater treatment* | toilet paper | Consumption of toilet paper in the wastewater treatment dataset of Food basket is overlapped with the use phase of the toilet paper in the Household goods basket. | Household goods | Because the use phase data of Household goods are more comprehensive. |
|  | wastewater | The same amount of water consumed in the use phase goes to wastewater treatment as sewage from residence. Hence, there is an overlapping also for wastewater treatment (as it is for water use) | Housing | Wastewater treatment is kept in BoP Housing for consistency with water use in the use phase |
|  | soap and detergent | Consumption of soap and detergent in the wastewater treatment dataset of basket Food are overlapped with the use phase of the soap and detergent in the Household goods and Appliances basket. | Household goods | Because the inventory data of soaps and detergents in BoP Household goods are more comprehensive. The data are from Ecolabel background studies. |
|  | electricity | Electricity consumption for the hand drying machine, and washing machine in the wastewater treatment dataset in Food basket are overlapped with the electricity consumption of the Housing. | Housing | Because of the same reason as for the use phase. |

## S4 Environmental impacts per person

Table S4.1 Impact by an average EU citizen, as calculated in each BoP and in the sum of the BoPs

| **Impact category** | **Unit** | **Housing** | **Mobility** | **Food** | **Appliances** | **Household goods** | **Sum of BoPs*** |
| --- | --- | --- | --- | --- | --- | --- | --- |
| Climate change | kg CO_2_ eq | 2.68E+03 | 2.49E+03 | 2.49E+03 | 3.48E+02 | 1.55E+03 | 9.55E+03 |
| Ozone depletion | kg CFC-11eq | 3.24E-04 | 5.80E-04 | 3.15E-03 | 8.82E-05 | 1.32E-04 | 6.41E-03 |
| Human toxicity, non-cancer | CTUh | 1.17E-04 | 8.99E-05 | 1.50E-04 | 3.10E-05 | 8.86E-05 | 5.00E-04 |
| Human toxicity, cancer | CTUh | 3.42E-05 | 2.65E-05 | 2.79E-05 | 7.04E-06 | 4.79E-05 | 1.42E-04 |
| Particulate matter | Disease incidence | 2.38E-04 | 9.88E-05 | 2.60E-04 | 1.00E-05 | 8.36E-05 | 7.32E-04 |
| Ionising radiation | kBq U^235^ eq | 2.02E+02 | 1.67E+02 | 5.08E+01 | 4.20E+01 | 9.27E+01 | 4.85E+02 |
| Photochemical ozone formation | kg NMVOC eq | 6.46E+00 | 1.02E+01 | 4.07E+00 | 8.95E-01 | 4.54E+00 | 2.60E+01 |
| Acidification | molc H^+^ eq | 1.32E+01 | 1.01E+01 | 3.54E+01 | 2.10E+00 | 8.45E+00 | 7.42E+01 |
| Eutrophication, terrestrial | molc N eq | 1.82E+01 | 3.00E+01 | 1.51E+02 | 3.01E+00 | 1.73E+01 | 2.51E+02 |
| Eutrophication, freshwater | kg P eq | 1.35E-01 | 7.26E-02 | 6.24E-01 | 7.89E-02 | 1.11E-01 | 9.28E-01 |
| Eutrophication, marine | kg N eq | 1.65E+00 | 2.74E+00 | 1.59E+01 | 3.78E-01 | 2.37E+00 | 2.34E+01 |
| Ecotoxicity, freshwater | CTUe | 1.12E+03 | 1.97E+03 | 6.69E+03 | 2.43E+02 | 1.96E+03 | 1.49E+04 |
| Land use | Pt | 5.63E+04 | 1.96E+04 | 2.41E+05 | 4.07E+03 | 5.91E+04 | 4.94E+05 |
| Water use | m^3^ world eq | 5.74E+03 | 4.37E+02 | 4.76E+03 | 1.55E+02 | 2.12E+03 | 1.40E+04 |
| Resource use, fossils | MJ | 4.77E+04 | 3.77E+04 | 1.45E+04 | 7.01E+03 | 2.28E+04 | 1.19E+05 |
| Resource use, mineral and metals | kg Sb eq | 5.05E-03 | 1.70E-02 | 2.31E-03 | 1.27E-02 | 5.94E-03 | 5.01E-02 |

*The impact of the overall consumption by an average EU-27 citizen is not equal to the sum of impact from single baskets because: 1) energy consumption in the use phase is totally accounted for in the use phase of housing to avoid double counting); 2) the quantity of food and appliances was upscaled to cover 100% of consumption by European citizens.

## S5 Impacts of single baskets in 2010 and 2015

The following paragraphs illustrate the impacts generated in 2010 and 2015 for each of the five areas of consumption (BoPs) considered. Results for the impacts generated at the European scale are reported in tables, whereas a set of graphs illustrates the variation per person, highlighting the contribution of the different product groups, housing types and means of transport.

**BoP Housing**

The reduction of energy use in the housing sector observed from 2010 to 2015 is reflected in the BoP Housing by a general decrease of impacts from 2010 to 2015, up to 10% in the impact categories to which electricity production was contributing the most in 2010 (e.g. ionising radiation) (Table S5.1).

*Table S5.1. Characterized results for the baseline scenario of BoP Housing in 2010 and 2015*

| **Impact category** | **Unit** | **2010** | **2015** | **Variation** |
| --- | --- | --- | --- | --- |
| Climate change | kg CO_2_ eq | 1.35E+12 | 1.23E+12 | -9% |
| Ozone depletion | kg CFC-11 eq | 1.63E+05 | 1.51E+05 | -7% |
| Human toxicity, non-cancer | CTUh | 5.87E+04 | 5.52E+04 | -6% |
| Human toxicity, cancer | CTUh | 1.72E+04 | 1.69E+04 | -2% |
| Particulate matter | Disease incidence | 1.20E+05 | 1.18E+05 | -2% |
| Ionising radiation | kBq U^235^ eq | 1.01E+11 | 9.12E+10 | -10% |
| Photochemical ozone formation | kg NMVOC eq | 3.25E+09 | 3.05E+09 | -6% |
| Acidification | molc H^+^ eq | 6.65E+09 | 6.14E+09 | -8% |
| Terrestrial eutrophication | molc N eq | 9.13E+09 | 8.64E+09 | -5% |
| Freshwater eutrophication | kg P eq | 6.78E+07 | 6.07E+07 | -10% |
| Marine eutrophication | kg N eq | 8.31E+08 | 7.86E+08 | -5% |
| Ecotoxicity freshwater | CTUe | 5.64E+11 | 5.46E+11 | -3% |
| Land use | Pt | 2.83E+13 | 2.74E+13 | -3% |
| Water scarcity | m^3^ water eq | 2.89E+12 | 2.96E+12 | 2% |
| Resource use (fossils) | MJ | 2.40E+13 | 2.17E+13 | -9% |
| Resource use (mineral and metals) | kg Sb eq | 2.54E+06 | 2.46E+06 | -3% |

*Figure S5.2. Comparison of impact of BoP Housing in the reference year 2010 and in 2015 (with total impact of year 2010 set as 100%) – split into the contributions of the various types of dwellings*


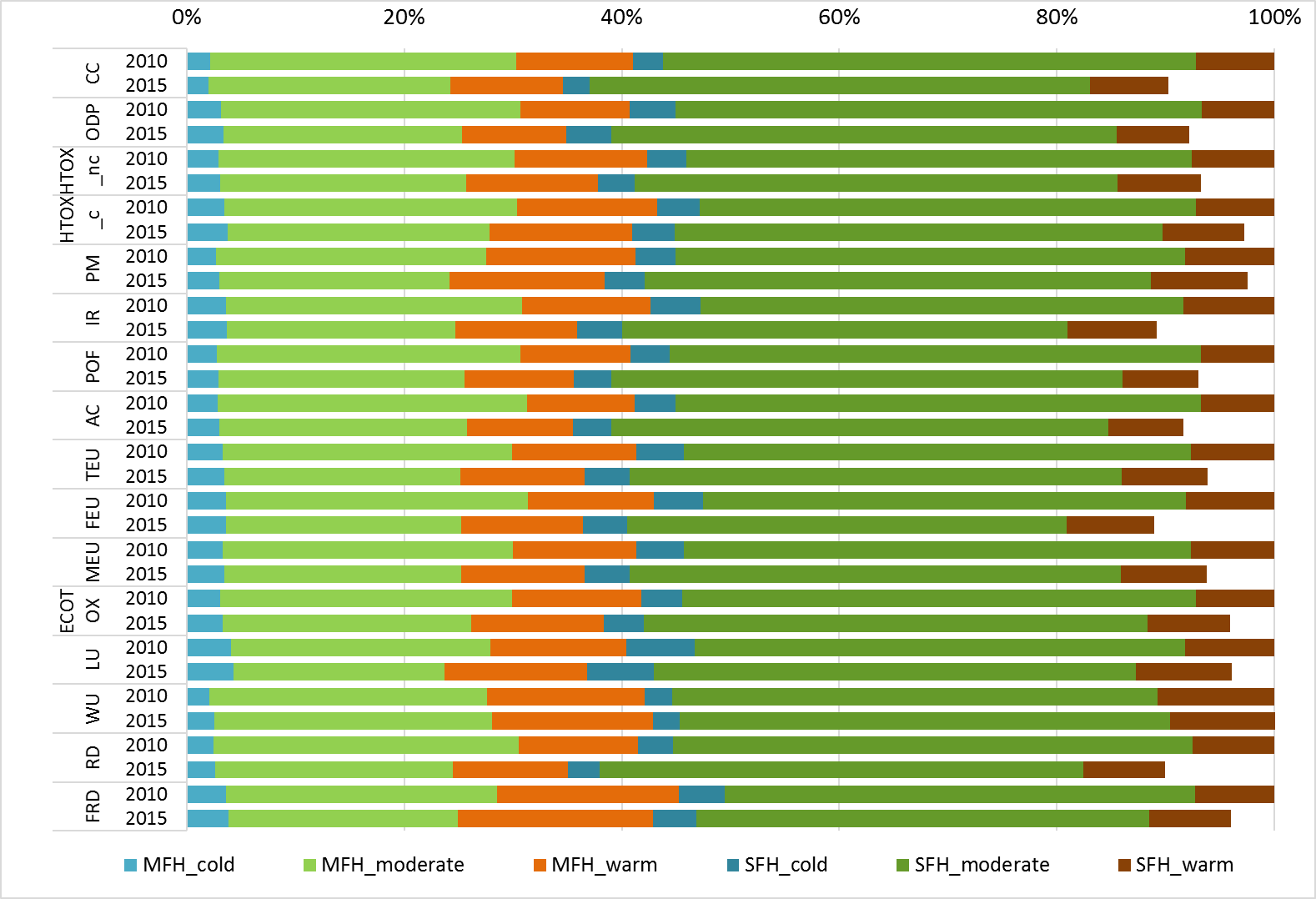


**BoP Mobility**

As illustrated in Table S5.3, the increase in the number of vehicles and pkm offsets, for most of the impact categories, the benefits generated by the introduction of Euro 6 vehicles and of hybrid and electric cars. The only two impact categories for which there is a reduction of impact are particulate matter (-4%) and photochemical ozone formation (-2%), mainly because of the reduced use of diesel cars.

*Table S5.3. Characterized results for the baseline scenario of BoP Mobility in 2010 and 2015*

| **Impact category** | **Unit** | **2010** | **2015** | **Variation** |
| --- | --- | --- | --- | --- |
| Climate change | kg CO_2_ eq | 1.25E+12 | 1.29E+12 | 3% |
| Ozone depletion | kg CFC-11 eq | 2.91E+05 | 3.00E+05 | 3% |
| Human toxicity, non-cancer | CTUh | 4.52E+04 | 4.58E+04 | 1% |
| Human toxicity, cancer | CTUh | 1.33E+04 | 1.34E+04 | 0% |
| Particulate matter | Disease incidence | 4.97E+04 | 4.79E+04 | -4% |
| Ionising radiation | kBq U^235^ eq | 8.38E+10 | 8.74E+10 | 4% |
| Photochemical ozone formation | kg NMVOC eq | 5.12E+09 | 5.03E+09 | -2% |
| Acidification | molc H^+^ eq | 5.09E+09 | 5.27E+09 | 4% |
| Terrestrial eutrophication | molc N eq | 1.51E+10 | 1.54E+10 | 2% |
| Freshwater eutrophication | kg P eq | 3.65E+07 | 3.75E+07 | 3% |
| Marine eutrophication | kg N eq | 1.38E+09 | 1.41E+09 | 2% |
| Ecotoxicity freshwater | CTUe | 9.89E+11 | 1.02E+12 | 3% |
| Land use | Pt | 9.86E+12 | 9.93E+12 | 1% |
| Water scarcity | m^3^ water eq | 2.19E+11 | 2.23E+11 | 2% |
| Resource use (fossils) | MJ | 1.90E+13 | 1.97E+13 | 4% |
| Resource use (mineral and metals) | kg Sb eq | 8.56E+06 | 8.53E+06 | 0% |

*Figure S5.4. Comparison of impact of BoP Mobility in the reference year 2010 and in 2015 (with total impact of year 2010 set as 100%) – split into the contributions of the various means of transport*


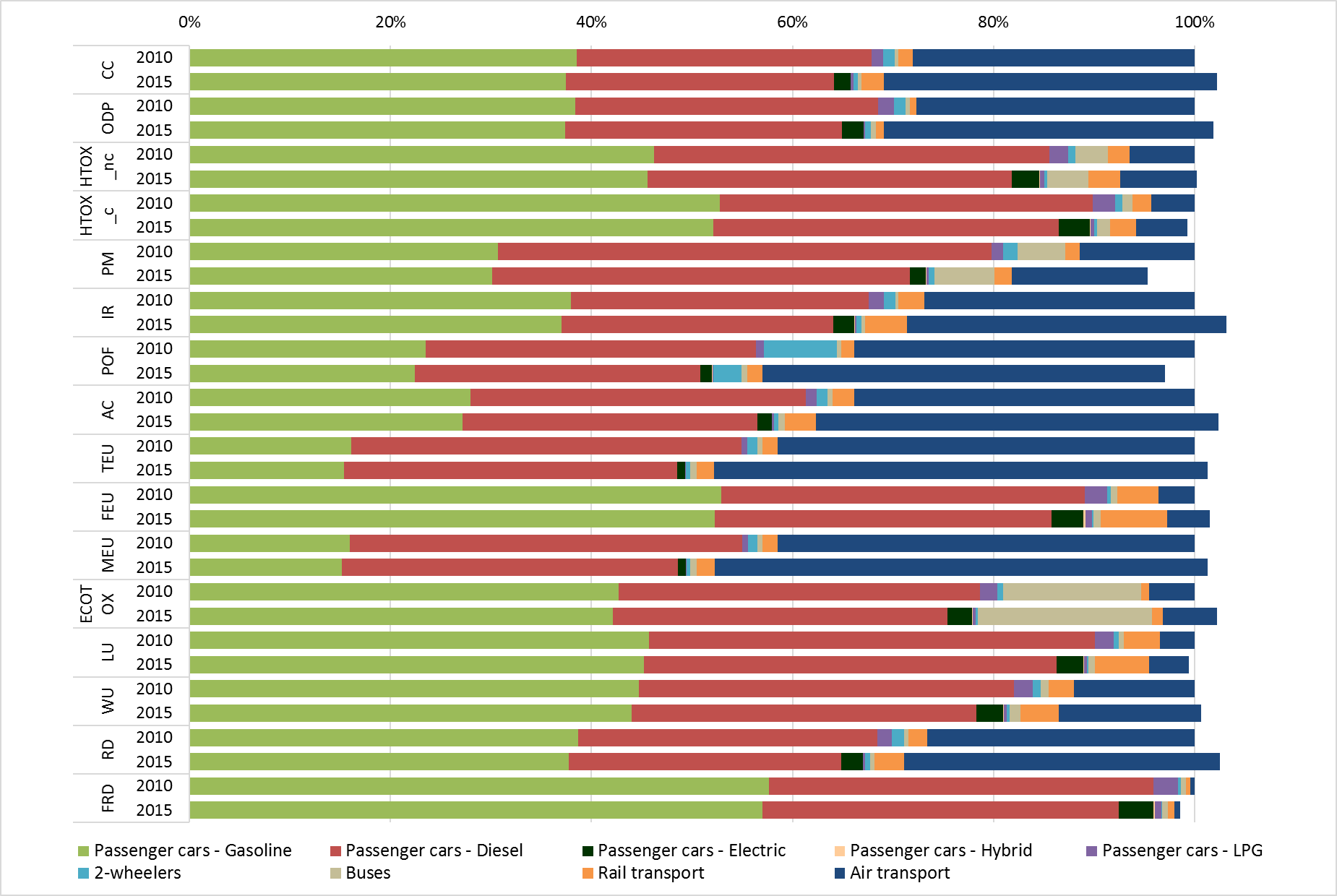


**BoP Food**

The impact generated in Europe by food consumption was generally higher in 2015 than in 2010, with the highest increase on photochemical ozone formation, on freshwater eutrophication and on ozone depletion (Table S5.5). The increase in photochemical ozone formation impact is mainly due to high increase in cod consumption, because cod fishing consumes significant amounts of diesel, causing photochemical ozone formation impact. In case of freshwater eutrophication, the main reason is high increase in salmon consumption, as nutrient emissions from salmon farming are high. Ozone depletion is mainly caused by the production and use of refrigerants during life cycle of some food products, including cod and salmon, which have the highest increase in consumption from 2010 to 2015, and thus being the main contributors to the increase of ozone depletion.

*Table S5.5. Characterized results for the baseline scenario of BoP Food in 2010 and 2015*

| **Impact category** | **Unit** | **2010** | **2015** | **Variation** |
| --- | --- | --- | --- | --- |
| Climate change | kg CO_2_ eq | 1.25E+12 | 1.38E+12 | 11% |
| Ozone depletion | kg CFC-11 eq | 1.58E+06 | 1.78E+06 | 12% |
| Human toxicity, non-cancer | CTUh | 7.55E+04 | 8.19E+04 | 9% |
| Human toxicity, cancer | CTUh | 1.40E+04 | 1.54E+04 | 10% |
| Particulate matter | Disease incidence | 1.30E+05 | 1.44E+05 | 10% |
| Ionising radiation | kBq U^235^ eq | 2.55E+10 | 2.72E+10 | 7% |
| Photochemical ozone formation | kg NMVOC eq | 2.04E+09 | 2.34E+09 | 14% |
| Acidification | molc H^+^ eq | 1.78E+10 | 1.97E+10 | 11% |
| Terrestrial eutrophication | molc N eq | 7.56E+10 | 8.37E+10 | 11% |
| Freshwater eutrophication | kg P eq | 3.14E+08 | 3.51E+08 | 12% |
| Marine eutrophication | kg N eq | 8.00E+09 | 8.91E+09 | 11% |
| Ecotoxicity freshwater | CTUe | 3.36E+12 | 3.68E+12 | 10% |
| Land use | Pt | 1.21E+14 | 1.33E+14 | 10% |
| Water scarcity | m^3^ water eq | 2.39E+12 | 2.59E+12 | 8% |
| Resource use (fossils) | MJ | 7.27E+12 | 7.94E+12 | 9% |
| Resource use (mineral and metals) | kg Sb eq | 1.16E+06 | 1.24E+06 | 7% |

*Figure S5.6 Comparison of impact of BoP Food in the reference year 2010 and in 2015 (with total impact of year 2010 set as 100%) – split into the contributions of the various product groups*


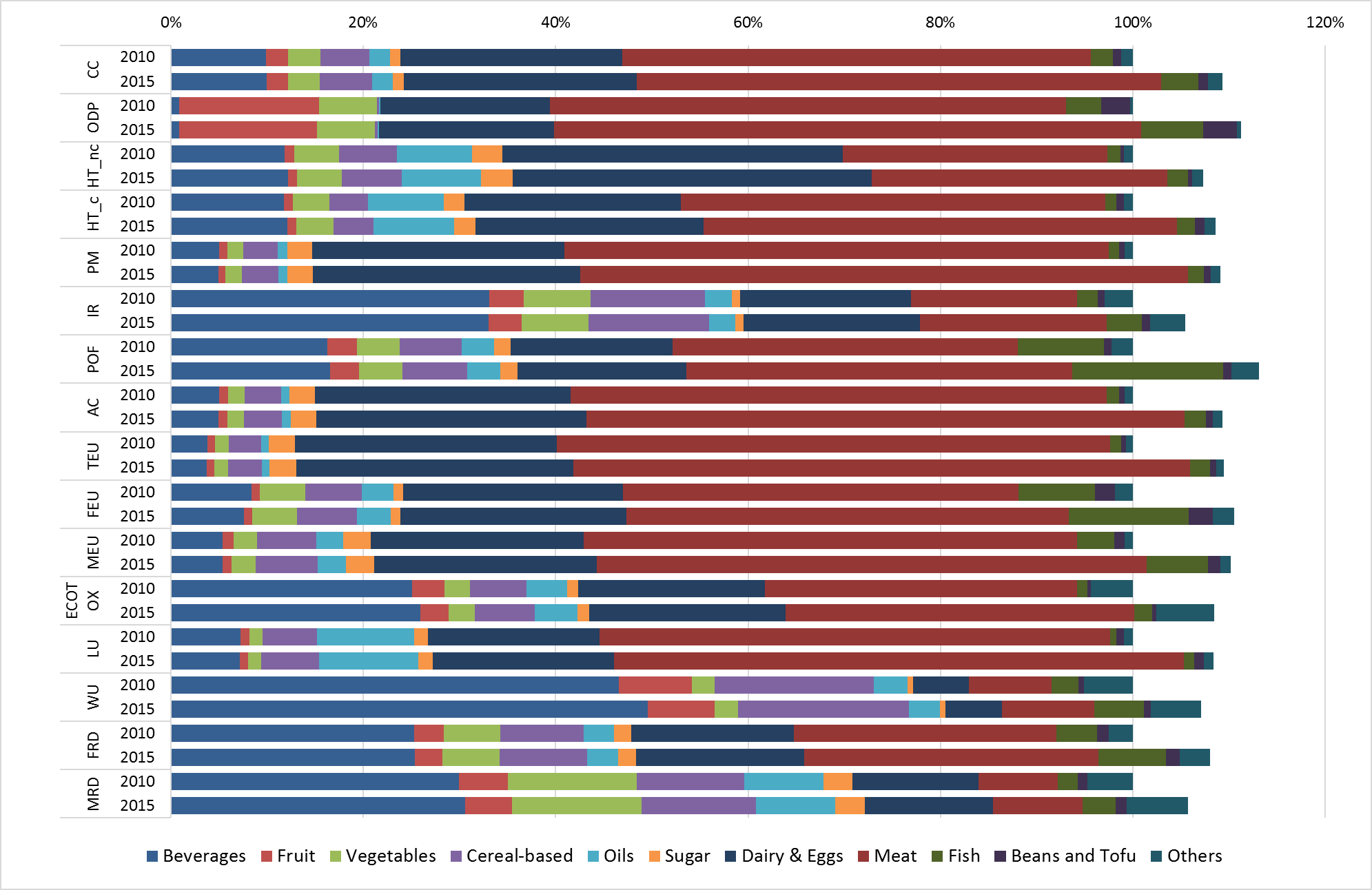


**BoP Appliances**

As a consequence of the general increase of apparent consumption of household appliances, the impact of BoP Appliances increased from 2010 to 2015 (Table S5.7). The highest increase is shown in the case of ozone depletion, due to the significant increase (53%) in the number of air conditioners owned by European households, and the related production and use of refrigerants. A significant increase (20%) occurs also for mineral resource depletion, due to the general increase in the number of devices and of resources (especially precious metals) used for their production.

*Table S5.7. Characterized results for the baseline scenario of BoP Appliances in 2010 and 2015*

| **Impact category** | **Unit** | **2010** | **2015** | **Variation** |
| --- | --- | --- | --- | --- |
| Climate change | kg CO_2_ eq | 1.75E+11 | 1.99E+11 | 14% |
| Ozone depletion | kg CFC-11 eq | 4.43E+04 | 6.25E+04 | 41% |
| Human toxicity, non-cancer | CTUh | 1.56E+04 | 1.81E+04 | 16% |
| Human toxicity, cancer | CTUh | 3.54E+03 | 3.94E+03 | 12% |
| Particulate matter | Disease incidence | 5.03E+03 | 5.79E+03 | 15% |
| Ionising radiation | kBq U^235^ eq | 2.11E+10 | 2.43E+10 | 15% |
| Photochemical ozone formation | kg NMVOC eq | 4.50E+08 | 5.16E+08 | 15% |
| Acidification | molc H^+^ eq | 1.05E+09 | 1.22E+09 | 15% |
| Terrestrial eutrophication | molc N eq | 1.51E+09 | 1.74E+09 | 15% |
| Freshwater eutrophication | kg P eq | 3.97E+07 | 4.68E+07 | 18% |
| Marine eutrophication | kg N eq | 1.90E+08 | 2.15E+08 | 13% |
| Ecotoxicity freshwater | CTUe | 1.22E+11 | 1.41E+11 | 15% |
| Land use | Pt | 2.05E+12 | 2.35E+12 | 15% |
| Water scarcity | m^3^ water eq | 7.81E+10 | 8.87E+10 | 14% |
| Resource use (fossils) | MJ | 3.52E+12 | 4.06E+12 | 15% |
| Resource use (mineral and metals) | kg Sb eq | 6.40E+06 | 7.69E+06 | 20% |

*Figure S5.8. Comparison of impact of BoP Appliances in the reference year 2010 and in 2015 (with total impact of year 2010 set as 100%) – split into the contributions of the various product groups*


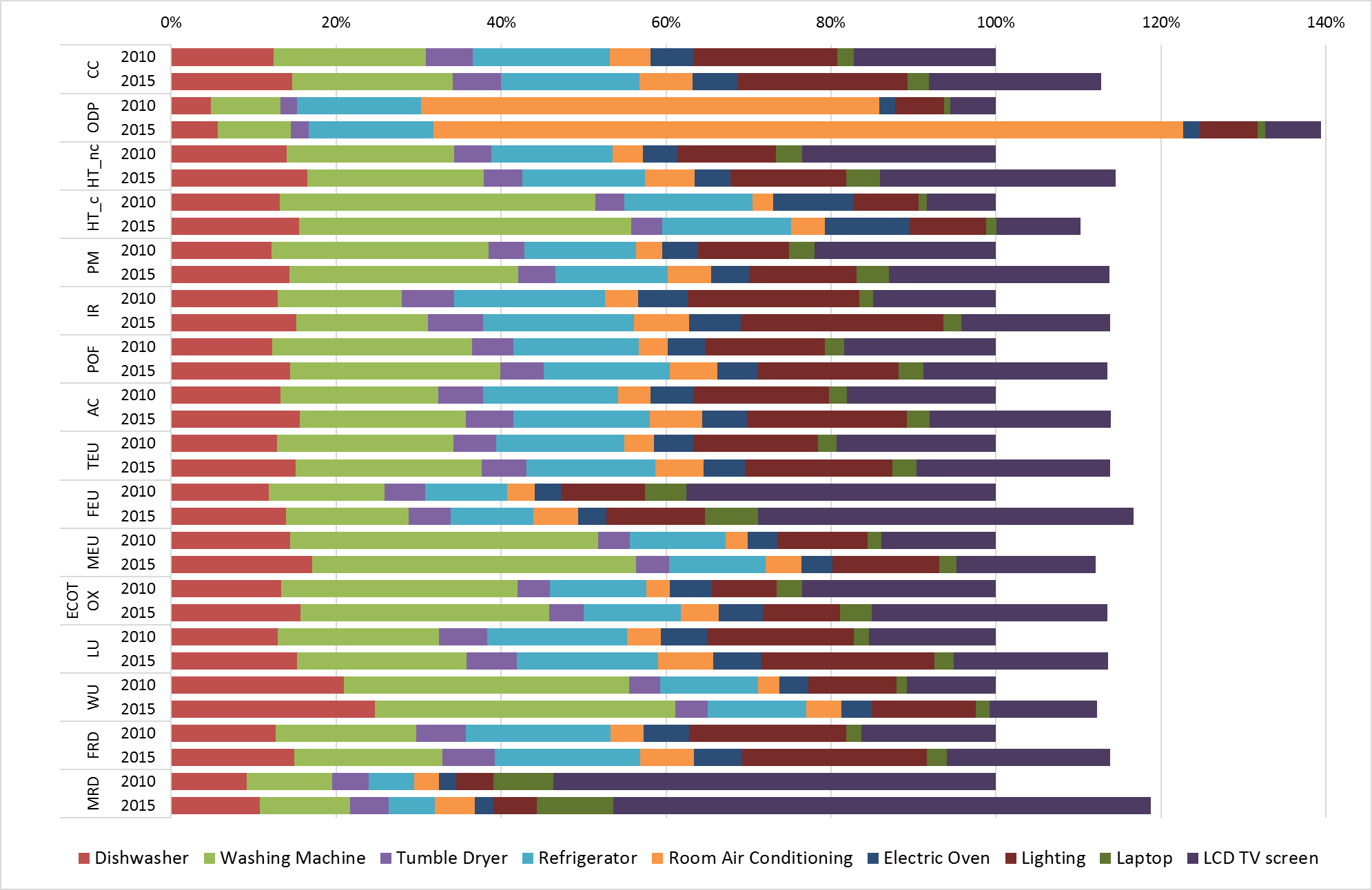


**BoP Household goods**

The combination of positive and negative variation of the quantity of products consumed is reflected in a quite limited variation of the impact generated by the consumption of household goods in Europe between 2010 and 2015. The characterized results of the BoP Household goods show a general increase in the impact generated, with the highest variation in water scarcity (14%), and human toxicity, non-cancer (11%) (Table S5.9).

*Table S5.9. Characterized results for the baseline scenario of BoP Household goods in 2010 and 2015*

| **Impact category** | **Unit** | **2010** | **2015** | **Variation** |
| --- | --- | --- | --- | --- |
| Climate change | kg CO_2_ eq | 7.77E+11 | 8.58E+11 | 10% |
| Ozone depletion | kg CFC-11 eq | 6.92E+04 | 7.33E+04 | 6% |
| Human toxicity, non-cancer | CTUh | 4.45E+04 | 5.09E+04 | -49% |
| Human toxicity, cancer | CTUh | 2.41E+04 | 2.52E+04 | 5% |
| Particulate matter | Disease incidence | 4.20E+04 | 4.60E+04 | 9% |
| Ionising radiation | kBq U^235^ eq | 4.66E+10 | 5.19E+10 | 11% |
| Photochemical ozone formation | kg NMVOC eq | 2.28E+09 | 2.50E+09 | 10% |
| Acidification | molc H^+^ eq | 4.25E+09 | 4.65E+09 | 10% |
| Terrestrial eutrophication | molc N eq | 8.71E+09 | 9.43E+09 | 8% |
| Freshwater eutrophication | kg P eq | 5.59E+07 | 6.22E+07 | 11% |
| Marine eutrophication | kg N eq | 1.19E+09 | 1.30E+09 | 9% |
| Ecotoxicity freshwater | CTUe | 9.84E+11 | 1.06E+12 | 8% |
| Land use | Pt | 2.97E+13 | 3.26E+13 | 10% |
| Water scarcity | m^3^ water eq | 1.06E+12 | 1.30E+12 | 22% |
| Resource use (fossils) | MJ | 1.14E+13 | 1.27E+13 | 11% |
| Resource use (mineral and metals) | kg Sb eq | 2.99E+06 | 3.28E+06 | 10% |

*Figure S5.10. Comparison of impact of BoP Household goods in the reference year 2010 and in 2015 (with total impact of year 2010 set as 100%) – split into the contributions of the various product groups*


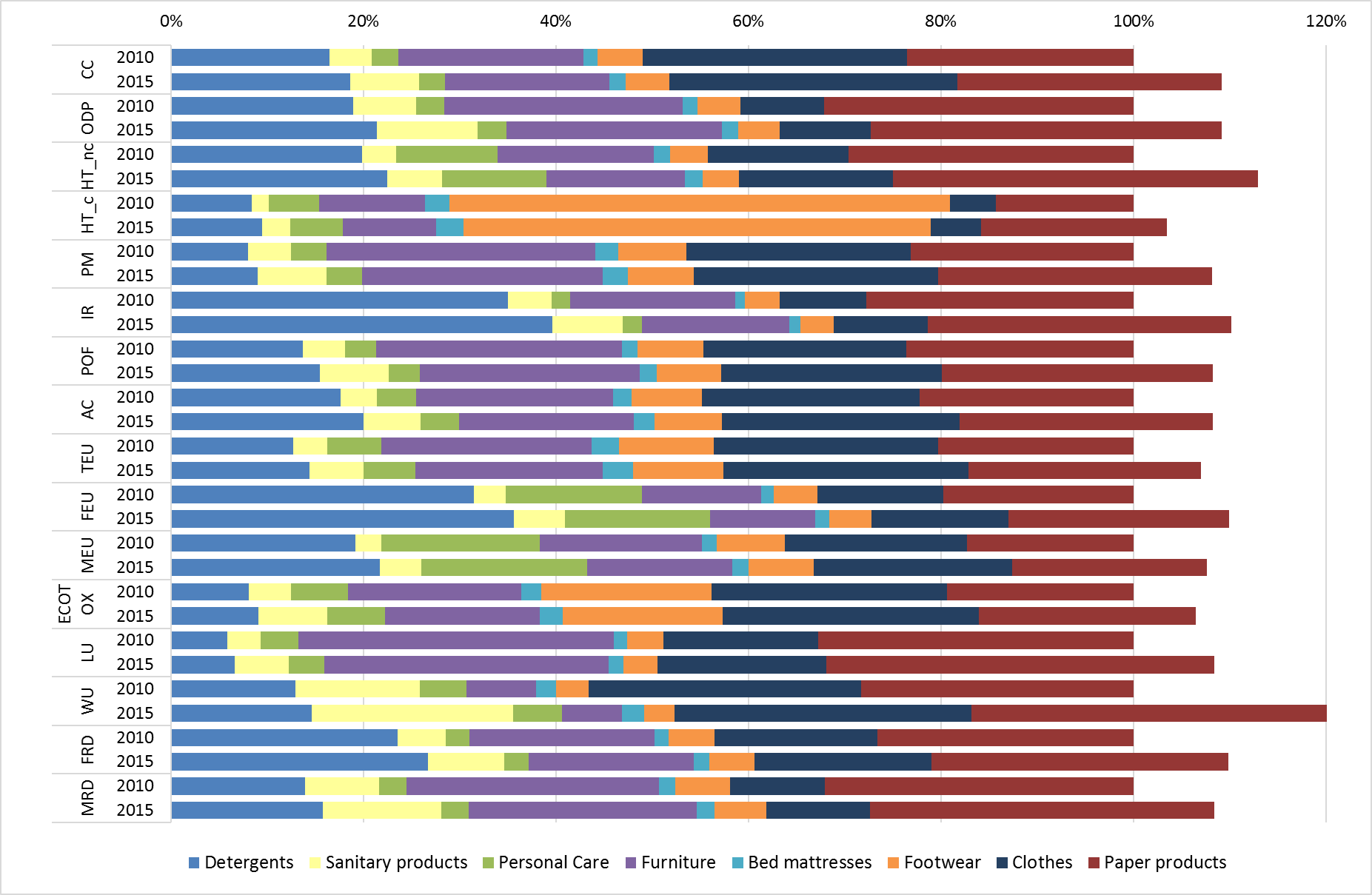


**Sum of BoPs**

When considering total household consumption, summing all five BoPs, it can be noted that environmental impacts increase in all impact categories (Table S2.12). The highest increase is in the mineral and metal resource use (10%).

*Table S5.11. Characterized results for the baseline scenario of total BoP in 2010 and 2015*

| **Impact category** | **Unit** | **2010** | **2015** | **Variation** |
| --- | --- | --- | --- | --- |
| Climate change | kg CO_2_ eq | 4.80E+12 | 4.97E+12 | 3% |
| Ozone depletion | kg CFC-11 eq | 3.22E+06 | 3.48E+06 | 8% |
| Human toxicity, non-cancer | CTUh | 2.51E+05 | 2.62E+05 | 4% |
| Human toxicity, cancer | CTUh | 7.14E+04 | 7.32E+04 | 3% |
| Particulate matter | Disease incidence | 3.68E+05 | 3.86E+05 | 5% |
| Ionising radiation | kBq U^235^ eq | 2.43E+11 | 2.46E+11 | 1% |
| Photochemical ozone formation | kg NMVOC eq | 1.30E+10 | 1.37E+10 | 5% |
| Acidification | molc H^+^ eq | 3.73E+10 | 3.96E+10 | 6% |
| Terrestrial eutrophication | molc N eq | 1.26E+11 | 1.37E+11 | 8% |
| Freshwater eutrophication | kg P eq | 4.66E+08 | 5.06E+08 | 8% |
| Marine eutrophication | kg N eq | 1.18E+10 | 1.28E+10 | 8% |
| Ecotoxicity freshwater | CTUe | 7.46E+12 | 8.03E+12 | 8% |
| Land use | Pt | 2.48E+14 | 2.64E+14 | 7% |
| Water scarcity | m^3^ water eq | 7.05E+12 | 7.31E+12 | 4% |
| Resource use (fossils) | MJ | 6.00E+13 | 6.09E+13 | 2% |
| Resource use (mineral and metals) | kg Sb eq | 2.52E+07 | 2.77E+07 | 10% |

## **References**

Bos U., Horn R., Beck T., Lindner J.P., Fischer M. (2016). LANCA® - Characterisation Factors for Life Cycle Impact Assessment, Version 2.0, 978-3-8396-0953-8Fraunhofer Verlag, Stuttgart (2016).

De Laurentiis V, Secchi M, Bos U, Horn R, Laurent A, Sala S (2019). Soil quality index: exploring options for a comprehensive assessment of land use impacts in LCA. J Clean Prod, 215, 63-74

EC (2017). PEFCR Guidance document - Guidance for the development of Product Environmental Footprint Category Rules (PEFCRs), version 6.3, December 2017.

Fantke, P., Evans, J., Hodas, N., Apte, J., Jantunen, M., Jolliet, O., McKone, T.E. (2016). Health impacts of fine particulate matter. In: Frischknecht, R., Jolliet, O. (Eds.), Global Guidance for Life Cycle Impact Assessment Indicators: Volume 1. UNEP/SETAC Life Cycle Initiative, Paris, pp. 76-99 (available at: www.lifecycleinitiative.org/applying-lca/lcia-cf/, accessed January 2017).

Frischknecht, R., Braunschweig, A., Hofstetter P., Suter P. (2000). Modelling human health effects of radioactive releases in Life Cycle Impact Assessment. Environmental Impact Assessment Review, 20 (2) pp. 159-189.

IPCC (2013). Myhre, G., D. Shindell, F.-M. Bréon, W. Collins, J. Fuglestvedt, J. Huang, D. Koch, J.-F. Lamarque, D. Lee, B. Mendoza, T. Nakajima, A. Robock, G. Stephens, T. Takemura and H. Zhang, 2013: Anthropogenic and Natural Radiative Forcing. In: Climate Change 2013: The Physical Science Basis. Contribution of Working Group I to the Fifth Assessment Report of the Intergovernmental Panel on Climate Change [Stocker, T.F., D. Qin, G.K. Plattner, M. Tignor, S.K. Allen, J. Boschung, A. Nauels, Y. Xia, V. Bex and P.M. Midgley (eds.)]. Cambridge University Press, Cambridge, United Kingdom and New York, NY, USA.

Posch, M., Seppälä, J., Hettelingh, J.P., Johansson, M., Margni M., Jolliet, O. (2008). The role of atmospheric dispersion models and ecosystem sensitivity in the determination of characterisation factors for acidifying and eutrophying emissions in LCIA. International Journal of Life Cycle Assessment (13) pp.477–486.

Rosenbaum, R.K., Bachmann, T.M., Gold, L.S., Huijbregts, M.A.J., Jolliet, O., Juraske, R., Köhler, A., Larsen, H.F., MacLeod, M., Margni, M., McKone, T.E., Payet, J., Schuhmacher, M., van de Meent, D., Hauschild, M.Z. (2008): USEtox - The UNEP-SETAC toxicity model: recommended characterisation factors for human toxicity and freshwater ecotoxicity in Life Cycle Impact Assessment. International Journal of Life Cycle Assessment, 13(7): 532-546, 2008.

Sala S., Crenna E., Secchi M., Pant, R., (2017) Global normalisation factors for the Environmental Footprint and Life Cycle Assessment, EUR (28984), Publications Office of the European Union, Luxembourg, 2017, ISBN 978-92-79-77214-6, doi 10.2760/775013

Sala, S., Benini, L., Castellani, V., Vidal Legaz, B., & Pant, R. (2018a). Environmental Footprint - Update of Life Cycle Impact Assessment methods. Resources, water, land and particulate matter. European Commission, Joint Research Centre.

Sala, S., Cerutti, A.K., & Pant, R. (2018b). Development of a weighting approach for Environmental Footprint. European Commission, Joint Research Centre, Publication Office of the European Union, Luxembourg. ISBN 978-92-79-68041-0

Struijs, J., Beusen, A., van Jaarsveld, H. and Huijbregts, M.A.J. (2009). Aquatic Eutrophication. Chapter 6 in: Goedkoop, M., Heijungs, R., Huijbregts, M.A.J., De Schryver, A., Struijs, J., Van Zelm, R. (2009). ReCiPe 2008 A life cycle impact assessment method which comprises harmonised category indicators at the midpoint and the endpoint level. Report I: Characterisation factors, first edition.

UNEP (2016). Global Guidance for Life Cycle Impact Assessment Indicators: Volume 1. UNEP/SETAC Life Cycle Initiative, Paris, pp. 76-99 (available at: www.lifecycleinitiative.org/applying-lca/lcia-cf/, accessed January 2019).

van Oers L, de Koning A, Guinee JB, Huppes G (2002): Abiotic Resource Depletion in LCA. Road and Hydraulic Engineering Institute, Ministry of Transport and Water, Amsterdam.

Van Zelm, R., Huijbregts, M.A.J., Den Hollander, H.A., Van Jaarsveld, H.A., Sauter, F.J., Struijs, J., Van Wijnen, H.J., Van de Meent, D. (2008). European characterization factors for human health damage of PM10 and ozone in life cycle impact assessment. Atmospheric Environment 42, 441-453.

WMO (2014). Scientific Assessment of Ozone Depletion: 2014. Global Ozone Research and Monitoring Project - Report No. 55, Geneva.

1. Available at: https://ec.europa.eu/energy/en/eu-buildings-database [↑](#footnote-ref-1)
2. IEE Project ENTRANZE (2014). Intelligent Energy Europe - Policies to ENforce the TRAnsition to Nearly Zero Energy buildings in the EU-27. Available at: http://www.entranze.eu (Accessed October 2014) [↑](#footnote-ref-2)
3. IEE Project TABULA (2012). Intelligent Energy Europe - Typology Approach for Building Stock Energy Assessment. Available at: http://webtool.building-typology.eu (Accessed October 2014) [↑](#footnote-ref-3)
4. IEE Project EPISCOPE (2012). Intelligent Energy Europe - Energy Performance Indicators for Building Stocks. Available at: http://episcope.eu (Accessed October 2014) [↑](#footnote-ref-4)
5. ODYSSEE-MURE (2015). Energy Efficiency Trends and Policies in the Household and Tertiary Sectors. Available at: http://www.odyssee-mure.eu/publications/br/energy-efficiency-trends-policies-buildings.pdf (Accessed May 2018) [↑](#footnote-ref-5)
